# Discussing Sexual Health During Diabetes Care, a Survey of UK Women—My Diabetes Nurse “Would Fall off Her Chair If I Mentioned It”

**DOI:** 10.3390/healthcare13212743

**Published:** 2025-10-29

**Authors:** Joanna Murphy, Debbie Cooke, David Andrew Griffiths, Emily Setty, Kirsty Winkley

**Affiliations:** 1 Faculty of Arts, Business, & Social Sciences, University of Surrey, Guildford GU2 7XH, UK; d.a.griffiths@surrey.ac.uk (D.A.G.); emily.setty@surrey.ac.uk (E.S.); 2 Faculty of Health & Medical Sciences, University of Surrey, Guildford GU2 7XH, UK; d.cooke@surrey.ac.uk; 3 Atlantis Health Ltd., Richmond TW9 1DL, UK; 4 Division of Care in Long-Term Conditions, Faculty of Nursing, Midwifery & Palliative Care, King’s College, London SE1 8WA, UK; kirsty.winkley@kcl.ac.uk

**Keywords:** diabetes mellitus, female sexual dysfunction, sexual health, women’s health, communication barriers, chronic disease

## Abstract

**Aims**: To ask UK women with diabetes whether they have discussed sexual health with healthcare professionals (HCPs) during diabetes care, and to explore communication barriers. **Methods**: An online questionnaire was developed, based on a published HCP communication survey, piloted by six women with diabetes. A total of 163 participants, recruited via social media and HCP network, completed Part 1 by selecting Likert or narrative response options, providing descriptive data. We report proportions with 95% confidence intervals (Wilson); percentages are calculated using the number responding to each item. Item-level missingness is retained as a non-analysed category, and the n is reported per question. No inferential comparisons were planned a priori. After Part 1 completion, participants could choose to finish, or to continue to Part 2 questions regarding vulval anatomy, function, and vocabulary (77 completed 2A: 80 completed 2B). Part 2 data was analysed thematically. **Results**: During diabetes care, a minority of participants, 44/163 (27%), said they had ever discussed sexual health, or had been advised how to access sexual health support, 28/163 (17%). If an HCP discussed sexual health, many women said they expected to feel surprised, 114/163 (70%), or pleased, 88/163 (54%). Some participants said they expected HCPs would find the topic inappropriate, 56/163 (36%), or annoying, 44/163 (27%). Some participants expressed HCP gender preference (75/163 [46%] female and 4/163 [3%] male) for such discussion. Part 2 findings revealed unmet sexual health literacy needs with potential to impact on communication with HCPs. **Conclusions**: Women reported infrequent communication about sexual health and diabetes during diabetes care. Findings highlight potential communication barriers for some participants including the following: unmet educational needs regarding diabetes and sexual health, lack of confidence about available support, fear of a negative HCP response, and preference for the gender of the HCP. Whereas in previous research, HCPs feared upsetting women by discussing sexual health, many participants said they expected to respond positively.

## 1. Introduction

Sexual health, including the potential for sexual enjoyment, is essential to overall health and identity (WHO, 2017) [1].

Research suggests that many women living with diabetes experience significant sexual health concerns; a narrative review showed that women living with diabetes, of any kind or duration, face high risk of female sexual dysfunction (FSD) and other sexual problems [2]. A systematic review found that FSD significantly impacts many women with Type 1 diabetes [3]. FSD refers to dissatisfaction with almost all sexual activity causing distress and lasting over 6 months [4]. Sexual problems are unlikely to be discovered by chance during routine health encounters, so proactive communication about sexual health is important for high-risk groups [5].

Research regarding communication about sexual health highlights the unmet need within women with diabetes. A survey of women with diabetes, of any kind, reported that a minority had sought help for sexual-health-related problems, and most were unaware that diabetes is linked to women’s sexual problems [6]. A survey of middle-aged and older adults showed that fewer women with diabetes (19%) had discussed sexual health with an HCP, compared to men (47%) [7], suggesting possible unmet needs for women. 

As well as the lack of awareness that diabetes is linked to sexual complications for women, research suggests discussing sex may generally be avoided as the topic is socially difficult to discuss; Montemurro et al. describe how sex, especially women’s sexual pleasure, may be avoided through fear of embarrassment and negative social judgement [8].

Zdilla describes linguistic difficulties as a barrier to discussing women’s sexual health, including avoidance of the term “vulva”, and comments that this linguistic avoidance potentially signals a sociocultural taboo [9]. Moreover, a survey suggested women in the general public have unmet educational needs regarding knowledge and language concerning the vulva [10]. Low levels of sexual health literacy have been linked to sexual dysfunction in diabetes [11], but research gaps remain regarding the link between sexual health literacy and communication about sexual problems in diabetes. In the research presented, exploratory questions regarding sexual health literacy themes are included. The vulva was chosen as an example sexual health topic when exploring participant’s linguistic and sexual health knowledge, since vulval infection, dryness, or lack of sensation are linked to diabetes complications, and because the vulva is one area of the body potentially linked to sexual enjoyment.

Expectations of receiving a negative response from an HCP in a clinical encounter may be relevant to the communication dynamic. In a narrative review of sexual health communication, Kingsberg relates women’s reticence to beginning conversations regarding sexual health to concerns about negative impact for HCPs [12], a theory which was echoed by some participants in Ejegi-Memeh’s qualitative work with women with type 2 diabetes [13].

Antos describes silence as a mutual communication dynamic, which only persists with the cooperation of all parties in the encounter [14]. In diabetes care, silence around women’s sexual health can be considered a “joint action” of everyone in the encounter [15,16]. 

The current survey focuses on women’s experience but is informed by the understanding of health communication as a joint action of HCPs and women, so this research builds on HCP research as well as studies of women with diabetes. In a recent survey of HCPs, most said they do not ask women about their sexual health during diabetes care [17]. HCPs mentioned inadequate training and time, and feared upsetting or offending women. HCPs wanted women to initiate conversations about sexual health, with some mentioning concerns that women would find the topic offensive or upsetting if introduced by an HCP, with a potential social risk for the HCP in starting the discussion. HCPs also expected women to prefer speaking to female HCPs about their sexual health, and some male professionals described the topic as difficult to discuss because of their gender. The current survey aims to explore whether women’s responses and concerns regarding communication are consistent with the HCPs’ expectations of their response [17], and vice versa. The current survey therefore asks women whether they agree with these HCP expectations of how they would respond to the topic, as well as exploring further potential communication barriers. With no validated survey tool yet available for this topic, the design of the survey was an iterative process, building on published, but not fully validated, survey tools used in previous research [10,17]. 

Madhu comments how the research community is also an actor in a dynamic which focuses on erectile dysfunction in diabetes sexual healthcare, overlooking women [18]. Silences at the level of individual clinical encounters risk in building wider patterns; under-identified complications are unlikely to be researched or mentioned in clinical guidelines. HCPs may not prioritise discussing a topic overlooked by clinical guidelines in future clinical encounters, and this can reinforce under-identification, risking marginalisation [19]. According to UK diabetes guidelines (NICE NG17 [last reviewed 19 September 2024] and NICE NG28 [last reviewed 23 December 2024]), based on the body of available research, clinicians should offer men, but not women, the opportunity to discuss their sexual health during routine diabetes care [20,21].

The aims of this research were to explore the following:

- Whether women have discussed sexual health during diabetes care, including their risk of sexual complications.
- Potential barriers to discussing sexual health during diabetes care, related to the following:
  - Women’s preferences regarding HCP gender
  - Practical barriers (including awareness of how to seek help, and having sufficient sexual health literacy and language to discuss the topic, taking the vulva as an exploratory example)
  - Women’s expectations of how discussing sexual health during diabetes care would impact on themselves or the HCP.

## 2. Methods

The study received ethical approval from the University of Surrey Ethics Committee (FASS 23-24 067 EGA).

### 2.1. Participant Inclusion Criteria

UK-resident women, aged 16+, and diagnosed with diabetes (any kind or treatment) by an HCP.

Self-identification as a woman, regardless of gender, sexuality, or sex at birth.

We included participants from the age of 16 years, because 16 is the legal age of consent for sexual activity in the UK, and because research, including qualitative interviews by Hoopes et al. [22], describes the importance for adolescents of communication about sexual health with clinicians. We included all ages above 16 because sexual healthcare is relevant for adults of all ages [1]. 

### 2.2. Survey Design

No validated survey exists regarding women’s experiences of communication concerning sexual health in diabetes care. 

Survey questions were developed from a published HCP survey on this topic, which had undergone steps in the validation process including establishing face validity and a think-aloud pilot phase (Part 1) [17], and from a published survey regarding public knowledge of vulval anatomy (Part 2) [10]. Further information about survey design and development, including the CHERRIES checklist, is available in Supplementary File S1.

Table 1 shows demographic data. Table 2 includes the Part 1 survey questions. Tables 3 and 4 include the Part 2 questions, with further information in Supplementary File S1.

The anonymous survey, hosted on Qualtrics (https://www.qualtrics.com, accessed on 1 November 2024) took approximately 15 min to complete via smartphone or computer. The data collection period was 1 November 2024 to 1 February 2025.

No participation incentive was given. The completed responses were eligible for a £100 voucher prize draw.

**Figure 1:**
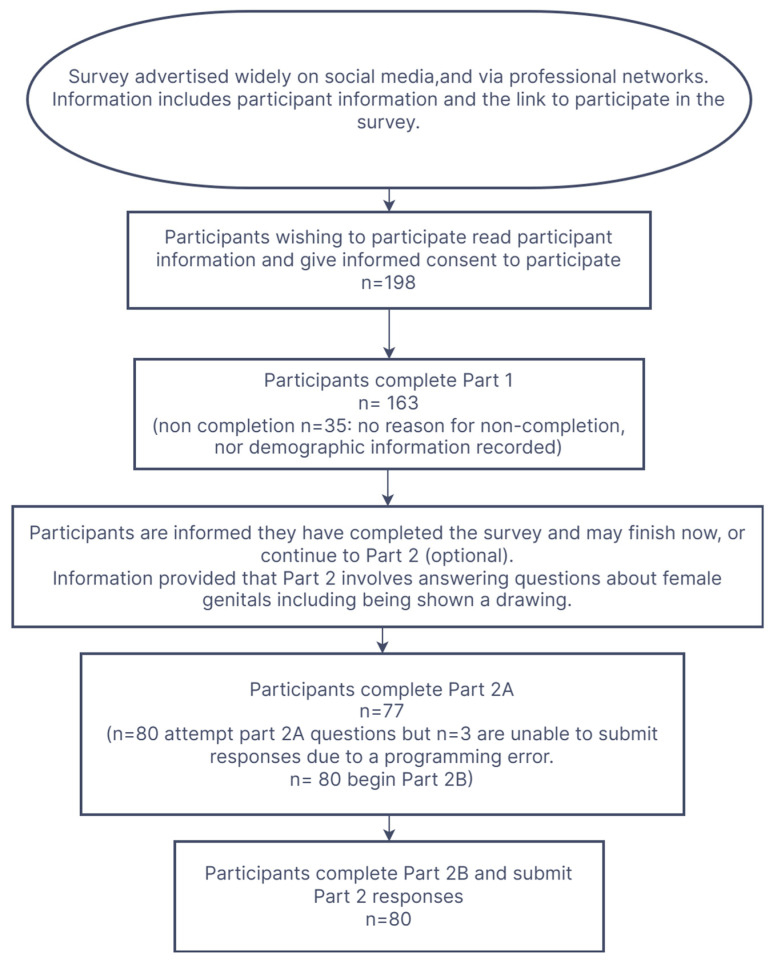
Survey Completion Flowchart.

The Survey Completion Flowchart (Figure 1), below, provides information about the completion pathway. 

### 2.3. Recruitment

The study was advertised on the Diabetes UK website, social media, Facebook, and Twitter pages aimed at women with diabetes (all types). The posts appeared in Facebook groups for Black British, Muslim, and Indian women. 

The survey was promoted to diabetes HCPs through informal professional networks and social media, including HCP-focused Facebook groups, Twitter, and LinkedIn.

The number and demographic information of potential participants who read invitations to participate but chose not to take part was not recorded, because of the nature of the open invitation and voluntary participation. 

Participants who clicked the link to express willingness to participate received information about the study (including opportunities to contact the researchers for further information, and signposting to resources related to the subject, including information and support). Those who then wished to volunteer to participate gave informed consent online. Supplementary File S1 (CHERRIES) provides further information about survey design, recruitment, participant information, consent, support, and ethical considerations.

### 2.4. Data Analysis

Descriptive statistics were conducted for participants characteristics, such as number and percentage (%), or mean and standard deviation (SD). We report proportions with 95% confidence intervals (Wilson), and percentages are calculated using the number responding to each item. Item-level missingness is retained as a non-analysed category, and the n is reported per question. No inferential comparisons were planned a priori. Data was analysed using SPSS (IBM SPSS Statistics V.30) and Excel (Microsoft Excel 2021). All participants completed questions 1–23, selecting responses from a Likert category, except questions 6–8, which offered narrative response options, developed in response to data from previous research as well as the literature review [17]. Total response numbers and blank responses are shown for each question. Participants could view questions but leave one or more question unmarked and move on. Because the study focuses on silences, in the results we consider blank responses to constitute a response, and we analyse and present the data of all 163 participants, including those who submitted a blank response to individual questions. Blank response numbers are recorded for each question. In Supplementary File S1, Supplementary Table S1 presents results with percentages calculated based on the total number of selected responses to the individual question, rather than based on all 163 participants (i.e., blank responses are not included). The responses were checked for straight lining/individual’s consistent blank responses, but no instances were detected. After question 23, participants could complete the survey or proceed to the optional Part 2 (2A and 2B). All participants were invited to comment on survey themes via a free-text box before completion.

Participants completing Part 2 answered question 2A where they were shown seven parts of the vulva using a labelled diagram and asked to identify the part by name or to describe what the part does, or to leave the answer blank if unknown) and 2B (typing words used for their vulva in three social contexts). Participants could leave answers blank and move on. Data were included in the analysis if participants pressed “finish survey and submit responses” after Part 1 or Part 2.

Part 2A responses were recorded and two independent researchers judged whether any entry typed by a participant was “correct” or “mis-identified”, following the methodology of the previously published survey on which Part 2A was based.

Answers accepted as “correct” gave either the anatomical or lay term for the body part, any phonetic spelling for this body part, or a description of the function of the body part. The rationale for the broad inclusion criteria for “correct” responses is that either a lay term, anatomical term, or simple description of the role of the body part would enable communication with an HCP.

For an answer to be classified as “mis-identified”, it either described the wrong function, or gave a function so generalised that it was judged by the two independent researchers that it would be unclear/misleading in an HCP conversation, or gave the wrong name for the part. 

Researchers made the “correct/mis-identified” allocation independently and met to compare and discuss any differences or difficulties in allocation. This discussion was required regarding decisions to accept as “correct” the answer “vulva” for specific parts of the vulva. The decision was made to accept as “correct” the term “vulva” for labia majora, labia minora, and perineum, because, whilst an over-generalisation which might need further clarification, the term would permit discussion of the connective tissue surrounding the opening of the vagina. The researchers decided not to accept the answer “vulva” as “correct” for vagina because communication misunderstanding is possible, since the vagina is an internal structure. The researchers decided not to accept the answer “vulva” for clitoris, because using a generalised anatomical term for this structure with a specific function and location was judged likely to impact negatively on communication, with potential for misunderstanding. Individual answers and groupings are presented to provide further information (Table 3), and coding matrix information is available in Supplementary File S1. 

Part 2B individual participant responses were recorded and coded by two independent researchers. The researchers followed a reflexive thematic approach [23], initially working independently to familiarise themselves with the data and generate codes, grouping data such that subthemes and themes emerged. The researchers met to discuss coding, grouping, and themes, and reached a consensus regarding any differences. Table 4 shows individual responses and themes. Supplementary File S1 provides further detail about the analysis. 

### 2.5. Free-Text Comments Thematic Analysis

Data were extracted from free-text “comments” available to all participants. Comments were coded and analysed by two independent researchers, using a reflexive thematic methodology, as in 2B [23]. Further detail is available in Supplementary File S1.

## 3. Results

A total of 198 women began the survey (excluding pilot participants), 163 completed Part 1, and 80 began Part 2.

Due to a programming error, the first three “Part 2” participants could not enter 2A answers and these data were excluded, but their other responses were unaffected. Programming was rectified for subsequent participants. Therefore, 77 responses were analysed for Part 2A, and 80 for Part 2B. All participants who began Part 2 completed Part 2B and submitted eligible responses. Participants could skip individual questions. Total response numbers are shown for each question in Table 2.

Table 1 shows participant demographic information.

Participants were recruited from across the UK, with 63.8% residing in England. UK population data allows comparison with survey demographics [23]. Characteristics for religious and ethnic group appear broadly consistent with overall UK population statistics, but Asian and Black ethnic groups may be under-represented, because of the increased diabetes prevalence in these populations [24]. Subgroup analysis was not included in survey aims and nor was judged appropriate due to survey size. In total, 80 (49%) participants reported living with Type 1 diabetes, 59 (36%) with Type 2 diabetes, and 6 (4%) reported living with other forms of diabetes/were unsure of diabetes type.

The women’s results were analysed together, regardless of type of diabetes, as women with diabetes of any kind or duration are at risk of sexual problems, which they may need to discuss with HCPs [2].

The mean participant age was 38 years (44 years for women with type 2 diabetes, 37 years for women with type 1 diabetes).

Part 1 survey results are shown in Table 2.

A minority of participants [44/163 (27%), Wilson CI 20.1–33.8%] had discussed sexual health during diabetes care. 

Most participants (114/163 [70%], Wilson CI 62.5–76.5%) would be surprised if an HCP addressed sexual health during diabetes care. 

Many women (91/163 [56%], Wilson CI 48.2–63.2%) felt uninformed about how diabetes affects women’s sexual health, and few reported that an HCP had discussed the increased risk of women’s sexual problems with diabetes (33/163 [20%], Wilson CI 14.8–27.1%). 

A minority of women (28/163 [17%], Wilson CI 12.2–23.7%) said they had been advised about whom to contact regarding sexual concerns. Regarding which HCP women would approach about sexual problems, the most frequently mentioned job roles were GPs (44/163 [27%]), walk-in sexual health clinics (20/163 [12%]), and diabetes secondary care professionals (18/163 [11%]). A total of 22/163 (14%) expected not to speak to any HCP.

Several participants expressed a preference regarding HCP gender when discussing sexual health, but this was not universal. One survey scenario (question 8) regarding preferred HCP gender resulted in 36/163 (22%, Wilson CI 16.4–29.1%) expressing no HCP gender preference, 75/163 (46%, Wilson CI 38.5–53.7%) expressing female HCP preference, 8/163 (5%, Wilson CI 2.5–9.4%) would not consult an HCP, and 4/163 (3%, Wilson CI 1.0–6.1%) expressing male HCP preference.

Regarding women’s expectations of being offered an opportunity to discuss sexual health information, 88/163 (54%, Wilson CI 46.3–61.5%) expected to be pleased, 40/163 (25%, Wilson CI 18.6–31.7%) embarrassed, 18/163 (11%, Wilson CI 7.1–16.8%] upset, 14/163 (9%, Wilson CI 5.2–13.9%) offended, and 23/163 (14%, Wilson CI 9.6–20.3%) forced to discuss an unwelcome subject. Several participants (89/163 [55%], Wilson CI 46.9–62.1%) felt this approach might result in their disclosing previously unmentioned concerns. A total of 114/163 (70%, Wilson CI 62.5–76.5%) participants expected HCPs consulted at diabetes check-ups to be trained about diabetes and women’s sexual problems. 

Regarding participants’ expectations of HCP response if women speak about sexual health during diabetes appointments, a minority (29/163 [18%], Wilson CI 12.7–24.4%) expected HCPs to feel pleased. Some participants expected HCPs to feel annoyed (44/163 [27%], Wilson CI 20.8–34.4%) or embarrassed (19/163 [18%], Wilson CI 12.9–24.6%). A total of 59/163 (36.2%, Wilson CI 29.2–43.8%) expected HCPs to consider the topic inappropriate. A minority (23/163 [14%], Wilson CI 9.6–20.3%) expected HCPs to consider sexual health a high priority, and few (17/163 [10%], Wilson CI 6.6–16.1%) expected HCPs to have time for the topic. A minority (26/163 [16%], Wilson CI 11.1–22.3%) expected good NHS treatments to be available for women’s sexual problems. A total of 68/163 ([42%], Wilson CI 34.4–49.4%) believed that women’s sexual enjoyment normally declines with age.

### 3.1. Survey Part 2 Results

#### 2A Result

Part 2A participants’ responses are shown in Table 3. 

The most frequently correctly identified structures were the vagina, labia majora and anus, each being correctly identified by 44/77 participants (57.1% of Part 2A participants, Wilson 95% CI 46–67.5%).

The least commonly correctly identified structures were the perineum (17/77 [22.1%], Wilson 95% CI 14.3–32.6%) and urethra (22/77 [28.6%], Wilson 95% CI 19.7–39.5%)

Mis-identifications occurred; for example, 19/77 (24.7%, Wilson 95% CI 16.3–35.4%) of 2A participants mis-identified the urethra as the clitoris. There were many blank responses, for example, 31/77 (40.3%, Wilson 95% CI 30.1–51.4%) of participants submitted a blank response when asked to identify the anus. 

Part 2B responses are shown in Table 4.

Part 2B responses highlighted a variety of different words, no words, or the uncertainty of words, used for the vulva, varying from individual to individual and according to social context. Some women described uncertainty regarding how to describe this body area to HCPs, and a strong theme emerged of using the word “vagina”. A minority of women, out of the 80 Part 2B participants, (7/80 [8.5%], Wilson CI 4.1–16.7%) mentioned using the word vulva with an HCP. 

When asked about words used for the vulva as a child, a theme emerged of never having used words for the vulva with carers, or having referred to the vulva by euphemism, including gastrointestinal euphemism such as “bottom or bum,” or having used a generalised incorrect anatomical term, often “vagina”. No participants remembered using the word “vulva”. Several women described using a word which would only be understood within their family (either made-up or rhyming words, i.e., “loo-loo”, “noo-noo”, or a term used idiosyncratically, out of usual context, i.e., “fairy” or “twinkle”).

In romantic and sexual relationships, themes emerged of women using the word “vagina” to mean “vulva”; some used no words, or were uncertain what word to use, several used a word related to female parts or to bits, and some used euphemisms related to “down below”. Some used familiar or vulgar terminology or words only understood within an individual relationship context.

The thematic analysis of the free-text comments is as follows:

A total of 18 participants provided free-text responses (excluding comments about survey design) regarding survey themes, ranging from 7 to 157 words. Responses were analysed thematically by two researchers working independently to familiarise themselves with the data, code, and group according to emergent themes and subthemes, before meeting to discuss themes and reach a consensus. Longer comments generated multiple codes and some fragments were coded to more than one theme. Further detail is available in Supplementary File S1. 

Four coherent themes emerged from coded data, as follows:

- Blood sugar management is the priority for Diabetes HCPs
- Diabetes HCPs discuss sex in connection to contraception and planning pregnancy rather than discussing positive sexual health or sexual enjoyment.
- Lack of awareness of a link between diabetes and sexual problems
- Silence/taboo regarding communication about sex during diabetes care.

Table 5 provides further information regarding free-text comments.

## 4. Discussion

This exploratory research asked women whether they had ever discussed sexual health during diabetes care and explored possible barriers to communication suggested by previous research. 

It highlights participants’ unmet sexual health needs. Despite being at a high risk of sexual problems related to diabetes, a minority (44/163 [27%], Wilson CI 20.1–33.8%) said they had ever discussed their sexual health with an HCP during diabetes care, and a minority (33/163 [20%], Wilson CI 14.6–26.8%) said they had been informed of a link between diabetes and possible sexual health complications, which could affect them. Regarding barriers to communication, sexual health literacy emerged as an avenue for future research. Many 2A participants asked to name (with non-specialist or anatomical words, or a simple description of what the body part does) seven parts of the vulva (vagina, labia majora, labia minora, clitoris, urethra, anus, and perineum), or to leave the answer blank if they did not know, did not provide an answer. Many words provided by Part 2A participants would not clearly convey meaning if used to discuss sexual health concerns or needs with an HCP or sexual partner. These findings suggest possible unmet sexual health literacy needs about the vulva, a body part related to sexual enjoyment and relevant for diabetes complications. These findings highlight how women at high risk of sexual health difficulties because of diabetes experience similar unmet sexual health literacy needs to the general population, concerning the vulva [10]. A lack of mutually understood language to articulate women’s sexual health experience and needs could be hypothesised to result in the topic being considered awkward or unpredictable to discuss, potentially contributing to avoidance. Further research on this hypothesis is needed. 

Future research could usefully focus on sexual health literacy needs in this high-risk group and explore whether specific unmet needs (and potentially addressing these) link to sexual health outcomes, including the use of validated assessment tools.

Some participants 68/163 (42%, Wilson CI 34.4–49.4%) said they expected sex to become less enjoyable with age, suggesting potential for the normalisation of the sexual complications of diabetes. Participants of all ages over 16 years old were included and analysed together in this exploratory survey. Future research could focus on the communication needs of women within specific age groups. Only 26/163 (16%, Wilson CI 11.2–22.3%) of participants said they believed good NHS treatments are available for women’s sexual problems; though, as Winkley describes, some diabetes sexual health complications, such as vaginal dryness or infection, or medication side effects, may be managed in primary care [2]. Women who believe sexual symptoms are to be expected, or are untreatable, may consider it futile to discuss difficulties with HCPs. The survey findings suggested many participants had received insufficient information about diabetes and sexual health to be able to understand and contextualise sexual complications and seek to help should they occur. Most participants believed HCPs had received adequate training to manage sexual health and diabetes, though the HCP survey [17] reported that HCPs were not confident in their knowledge and skills of diabetes and women’s sexual health, which was a communication barrier for HCP participants. 

Most participants (114/163 [70%], Wilson CI 62.5–76.5%) said they anticipated surprise if HCPs raised the issue, suggesting this would challenge norms in diabetes care. Free-text comments provide further detail on the degree of surprise anticipated, including a free-text quote that the topic would cause a diabetes nurse to “fall off her chair.”

Surprise, however, seems not to equate to finding the topic universally unwelcome. Contrary to previously reported HCP concerns that most women would respond negatively to speaking about sexual health [17], the survey results suggest many participants would welcome the opportunity for information and discussion.

Challenging expectations by acting differently from usual patterns of behaviour risks incurring negative social consequences [25]. The existing norms of what is discussed during diabetes care may shape the themes women and HCPs consider appropriate to discuss. Future research could explore communication approaches aimed at introducing the topic of sexual health acceptably and signalling that the topic can be spoken about in this context. The PLISSIT model [26] is one such approach, consisting of the following stepwise:

- *Permission* (P) to discuss sexual health during the consultation gained by the non-specialist HCP. An example could include saying, “We are giving information to everyone who comes to the clinic today about sexual health and diabetes. Is it OK with you to talk about this subject?”
- *Limited Information* (LI) about sexual health provided by the non-specialist HCP. An example includes saying, “Research shows women who live with diabetes are at increased risk of experiencing sexual problems. Here is more information about sexual health and diabetes [provide information link or leaflet]. You can talk to me or your GP [or other HCP as appropriate to local services] about sexual health and diabetes. Would you like to discuss any concerns today?”
- *Specific Suggestions* (SS) given, based on managing the individual woman’s concerns, or accessing further support, according to existing HCP training and competence. An example is a primary care HCP advising a woman to use lubricants for vaginal dryness, providing this is within the HCP’s existing professional scope and competence, or signposting the woman to a colleague who can help.
- *Intensive Therapy* (IT) offered, which may include specialist management. An example includes making a referral for psychosexual counselling.

PLISSIT was acceptable to women in a previous diabetes research study [27]. The initial steps of PLISSIT do not require specialist sexual health training and could include measures as simple as asking women if they would like information about women’s sexual health and diabetes. Despite its simplicity, this question would potentially challenge a complex social dynamic of silence, and signal that sexual health is part of women’s diabetes care. Future research could pilot interventions such as seeking advance permission to discuss sexual health during routine reviews.

However, in the HCP survey [17], the lack of HCP training and perceived inadequate clinical skills to cope, should women discuss sexual symptoms, was described as a barrier to HCPs starting discussions. Although no specialist training on women’s sexual health and diabetes is required in order to signpost women to existing health information and services, future research could explore whether training impacts on the care provided and on women’s outcomes. 

Many women expected HCPs would find the topic of sexual health unwelcome during diabetes care, which could be a significant communication barrier. A minority of participants said they expected HCPs to feel pleased (29/163 [18%], Wilson CI 12.9–24.6%), with some (59/163 [36%], Wilson CI 29.0–43.6%) expressing concern HCPs would consider the topic inappropriate. Some women (81/163 [50%], Wilson CI 42.4–57.6%) commented HCPs have insufficient time for this topic, which they did not expect to be considered a high priority for HCPs (84/163 [52%], Wilson CI 44.4–59.5%). Some participants (44/163 [27%], Wilson CI 20.8–34.3%) expected HCPs to feel annoyed if women started discussing sexual concerns. 

Ironically, many participants in the HCP survey reported they would willingly discuss sexual health during diabetes care if a woman raised the subject, though they did not want to start the conversation themselves [17]. In this survey, HCPs expressed concern about upsetting women by introducing an unwelcome topic into the conversation. Many HCPs expected women would not wish to speak to a male HCP. Approximately half of the women in the current survey expressed a gender preference, mostly for a female HCP, regarding discussing sexual health. More research is needed regarding the meaning of this gender preference, and most acceptable ways for HCPs providing diabetes care to respond. 

Together, the two surveys describe a sense of mutual hesitation to begin discussions, based partly on fears of causing offence to the other, which seems to not always be warranted. This hypothesis would benefit from further study.

The strengths of this study are that we designed a questionnaire to investigate a gap in research concerning communications and silences about women’s sexual health during diabetes care. There was no pre-existing fully validated tool, but the survey design was an iterative process, including building upon two published surveys, one of which has undergone steps in the validation process. The absence of a fully validated tool specifically designed to assess women’s experiences of sexual health communication in diabetes care represents a limitation of this study. Future research should prioritise the development and validation of such instruments to strengthen methodological rigour and comparability across studies.

The exploratory findings of this survey provide a foundation for future in-depth studies into this complex communication dynamic. Qualitative studies would be a valuable next step to explore, in depth, women’s perspectives regarding discussing sexual health with HCPs during diabetes care.

Participants are broadly representative of the local UK population but the response rate amongst the non-white population was lower than the background population of diabetes, suggesting potential under-representation, which has been documented in previous studies [28]. Given participation in this survey is voluntary, and relied predominantly on social media platforms, those for whom the topic is most unacceptable, or less well-known, may not have participated. Online recruitment and completion may have biassed the sample group towards women who engage with online technology. This may have introduced a selection bias, as women who are more engaged, and health- and IT-literate, or comfortable discussing sexual health might have been more likely to participate and express opinions. This could reduce the generalisability of results to women with diabetes from groups which are under-represented, as well as the generalisability to women who are less digitally connected, or less willing to engage with this topic. Further research is needed, which could include purposively sampling diverse populations. A future study could involve asking women whether they are willing to receive information regarding sexual health and diabetes, and could record the demographic information for women who decline this offer. This would provide information regarding selection bias, which is difficult to obtain from this voluntary study. This survey relies on self-report and includes some questions regarding expected responses to hypothetical scenarios, which many participants had not experienced. Responses are therefore subject to bias, including self-report and social desirability bias; further in-depth research is needed.

Given the paucity of other research and the limitations regarding the generalisability of these findings to under-represented groups, further research is needed before detailed recommendations for clinical practice can be made. However, in the interests of good care, HCPs working with women with diabetes could usefully undertake the following:

- Participate in professional education about diabetes and women’s sexual health to ensure competent to provide basic health education on this topic and signpost to support.
- Include the “permission” and “limited information” stages of PLISSIT during routine women’s diabetes care.

## 5. Conclusions

This exploratory study reveals that most participants had not discussed sexual health during care, had not been informed of the link between diabetes and women’s sexual complications, and had not been advised how to access support for sexual health. Multiple barriers to communication were identified, including concern that HCPs do not wish to discuss this topic, and participants’ lack of awareness about women’s sexual complications of diabetes.

While a minority of participants reported expecting to feel upset or offended by discussing the topic, many would be pleased. These results should be treated cautiously as participation was voluntary and those most upset or offended by the subject may have declined to take part. Many participants reported that such discussions would be surprising. Given the sociocultural challenge of discussing an unexpected theme during busy healthcare encounters, and the multiple other actual and expected communication barriers identified, it seems unrealistic to expect women to “speak up” first about sexual health during routine diabetes care. Providing reassurance that HCPs are open to this conversation may be a useful first step in addressing a communication gap. The use of approaches, such as PLISSIT, to begin conversations about sexual health acceptably in women’s diabetes care would benefit from further research. 

This study highlights the need for greater attention to women’s sexual health in diabetes care, including informing women about the potential sexual complications of diabetes and how to seek support. 

## Figures and Tables

**Table 1:** Demographic characteristics of participants (n = 163).

Participant Category	Number of Responses (% of All Part 1 Participants) or Mean (SD)
Total participants All participants identify as women. Gender and sex category information: Identify as women 163 (100%) Identify as non-binary 2 (1.2%) Commented they had been assigned male sex at birth 2 (1.2%)	163 (100%)
Country in which participant currently lives	Total number of responses 143 (87.7%)
	Blank responses 20 (12.3%)
England	104 (63.8%)
Scotland	8 (4.9%)
Wales	13 (8.0%)
Northern Ireland	18 (11.0%)
Type of diabetes	Total responses 145 (89.0%)
	Blank responses 18 (11.0%)
Type 1	80 (49.1% Wilson CI 41.5–56.6%)
Type 2	59 (36.2% Wilson CI 29.2–43.8%)
Other (including “do not know”, or “other type of diabetes”)	6 (3.7%)
Religious background (where specified)	Total responses 121 (74.2%)
	Blank responses 42 (25.8%)
No religion	57 (35.0%)
Buddhist	2 (1.2%)
Christian (all categories)	49 (30.1%)
Hindu	6 (3.7%)
Jewish	1 (0.6%)
Muslim	5 (3.1%)
Sikh	1 (0.6%)
Average age	Total responses 110 (67.5%)
	Blank responses 53 (32.5%)
All participants mean age (years)	38.4 (SD 14.0)
Number of participants in age group of 16–24	32 (19.6%)
Number of participants in age group of 25–34	26 (16.0%)
Number of participants in age group of 35–44	11 (6.7%)
Number of participants in age group of 45–54	23 (14.1%)
Number of participants in age group of 55–64	14 (8.6%)
Number of participants in age group of 65 and over	4 (2.5%)
Average age of women with Type 1 Diabetes (where specified)	36.6 years (SD 14.5, Median 30, IQR 29)
Average age of women with Type 2 Diabetes (where specified)	43.6 years (SD 12.9, Median 44.5, IQR 24.5)
Ethnic group (where specified)	Total responses 125 (76.7%)
	Blank responses 38 (23.3%)
White British	79 (48.5%)
White Irish	8 (4.9%)
White Other	16 (9.8%)
Mixed/Multiple ethnic groups:	
White and Asian	5 (3.1%)
White and Black African	5 (3.1%)
Indian	5 (3.1%)
Black/African/Caribbean/Black British	1 (0.6%)
Chinese	2 (1.2%)
Pakistani	3 (1.8%)
Other ethnic group (unspecified)	1 (0.6%)

**Table 2:** Main survey question responses (number and percentage of respondents).

Question Stem (% of 163 Participants)	Response Options (% of 163 Participants)						
	Strongly Agree	Somewhat Agree	Neither Agree Nor Disagree	Somewhat Disagree	Strongly Disagree	Do not Know	
At a diabetes check-up, if a health professional asks about my sex life, I would be surprised. Total question responses: 143 Blank responses: 20 (12.3%)	88 (54.0%)	26 (16.0%)	11 (6.7%)	9 (5.5%)	7 (4.3%)	2 (1.2%)	
2. I have discussed my sex life at a diabetes check-up in the past. Total question responses: 139 Blank responses: 24 (14.7%)	17 (10.4%)	27 (16.6%)	9 (5.5%)	20 (12.2%)	65 (40.0%)	1 (0.6%)	
3. I feel well-informed about how diabetes can affect women’s sex lives. Total question responses: 157 Blank responses: 6 (3.7%)	21 (12.9%)	21 (12.9%)	10 (6.1%)	25 (15. 3%)	66 (40.5%)	14 (8.6%)	
4. A healthcare professional has told me women with diabetes are at increased risk of having problems with their sex lives. Total question responses: 141 Blank responses: 22 (13.5%)	22 (13.5%)	11 (6.7%)	11 (6.7%)	19 (11.7%)	73 (44.8%)	5 (3.1%)	
5. I have been advised who to speak to about my sex life if I have any concerns. Total question responses: 146 Blank responses: 17 (10.4%)	12 (7.4%)	16 (9.8%)	13 (8.0%)	19 (11.7%)	83 (50.9%)	3 (1.8%)	
6. If I had a problem with my sex life, the healthcare professional I would talk to first is… Total question responses: 128 Blank responses: 35 (21.5%)	Response options			Number of responses (% of all participants)			
	GP			44 (27.0%)			
	Practice nurse			9 (5.5%)			
	Walk in sexual health clinic			20 (12.3%)			
	Diabetes secondary care team professional			18 (11.0%)			
	I probably would not speak to a health professional at all			22 (13.5%)			
	This situation would never be relevant to me			2 (1.2%)			
	Do not know			11 (6.7%)			
	Other			2 (1.2%) Free-text responses: “peer support group”; “If I knew it was diabetes related, I would speak to the diabetes team, otherwise I would speak to the GP or sexual health clinic”			
7. If needed help with problems with my sex life, and had to make an appointment with someone I hadn’t met before, I would prefer to speak to… Total question responses: 113 Blank responses: 50 (30.7%)	Response options						
	A female HCP	A male HCP	An HCP of any gender	Do not know	I wouldn’t speak to an HCP	This subject would never be relevant to me	An HCP who identifies as non-binary or gender queer
	60 (36.9%)	5 (3.1%)	35 (21.5%)	4 (2.5%)	6 (3.7%)	1 (0.6%)	2 (1.8%)
8. You have known two healthcare professionals well for a long time. They have the same job and are the same age. You trust them both equally. They both listen well and have good knowledge. As far as you can tell from appearance and name, one professional is a man, and one is a woman. Please consider the following statement “If I had problems with my sex life, I would prefer to speak to… ” Total question responses: 126 Blank responses: 37 (22.7%)	Response options						
	The female HCP	The male HCP	Either HCP	I do not know	I wouldn’t speak to an HCP		
	75 (46.0%)	4 (2.5%)	36 (22.1%)	3 (1.8%)	8 (4.9%)		
9. For me personally: At a diabetes check-up, if I am offered information about sex and diabetes and the option to discuss this topic, I will feel pleased. Total question responses: 130 Blank responses: 33 (20.2%)	Response options						
	Strongly Agree	Somewhat agree	Neither agree nor disagree	Somewhat disagree	Strongly disagree	Do not know	
	47 (28.8%)	41 (25.1%)	22 (13.5%)	10 (6.1%)	4 (2.5%)	6 (3.7%)	
10. For me personally: At a diabetes check-up, if I am offered information about sex and diabetes and the option to discuss this topic, I will feel upset. Total question responses: 132 Blank responses: 31(19.0%)	12 (7.4%)	6 (3.7%)	9 (5.5%)	18 (11.0%)	84 (51.5%)	3 (1.8%)	
11. For me personally: At a diabetes check-up, if I am offered information about sex and diabetes and the option to discuss this topic, I will feel offended. Total question responses: 135 Blank responses: 28 (17.1%)	5 (3.1%)	9 (5.5%)	7 (4.3%)	6 (3.7%)	105 (64.4%)	3 (1.8%)	
12. For me personally: At a diabetes check-up, if I am offered information about sex and diabetes and the option to discuss this topic, I will feel embarrassed. Total question responses: 138 Blank responses: 25 (15.3%)	11 (6.7%)	30 (18.4%)	16 (9.8%)	27 (16.6%)	49 (30.0%)	5 (3.1%)	
13. For me personally: At a diabetes check-up, if I am offered information about sex and diabetes and the option to discuss this topic, I might mention problems I haven’t spoken to healthcare professionals about yet. Total question responses: 132 Blank responses: 31 (19.0%)	35 (21.5%)	54 (33.1%)	12 (7.4%)	11 (6.7%)	14 (8.6%)	6 (3.7%)	
14. For me personally: At a diabetes check-up, if I am offered information about sex and diabetes and the option to discuss this topic, I would feel forced to discuss something I would rather not talk about. Total question responses: 151 Blank responses: 12 (7.4%)	6 (3.7%)	17 (10.4%)	16 (9.8%)	16 (9.8%)	92 (56.4%)	4 (2.5%)	
15. I expect the health professionals I see at diabetes check-ups are trained about diabetes and women’s sexual problems. Total question responses: 156 Blank responses: 7 (4.3%)	72 (44.2%)	42(25.8%)	13 (8.0%)	9(5.5%)	9 (5.5%)	11 (6.7%)	
16. If I start talking about my sex life at a diabetes check-up, I expect the health professional will feel pleased. Total question responses: 129 Blank responses: 34 (20.9%)	11 (6.7%)	18 (11.0%)	23 (14.1%)	23 (14.1%)	46 (28.2%)	8 (4.9%)	
17. If I start talking about my sex life at a diabetes check-up, I expect the health professional will think the topic is inappropriate. Total question responses: 136 Blank responses: 27 (16.6%)	38 (23.3%)	21 (12.9%)	21 (12.9%)	15 (9.2%)	29 (17.8%)	12 (7.4%)	
18. At a diabetes check-up, my sex life is a high priority for the health professional. Total question responses: 123 Blank responses: 40 (24.5%)	10 (6.1%)	13 (8.0%)	11(6.7%)	14 (8.6%)	70 (42.9%)	5 (3.1%)	
19. At a diabetes check-up the healthcare professional has time to discuss my sex life. Total question responses: 133 Blank responses: 30 (18.4%)	12 (7.4%)	15 (9.2%)	18 (11.0%)	27 (16.6%)	54 (33.1%)	7 (4.3%)	
20. If I start talking about my sex life at a diabetes check-up, I expect the healthcare professional will feel annoyed. Total question responses: 109 Blank responses: 54 (33.1%)	25 (15.3%)	19 (11.7%)	11 (6.7%)	19 (11.7%)	30 (18.4%)	5 (3.1%)	
21. If I start talking about my sex life at a diabetes check-up, I expect the healthcare professional will feel embarrassed. Total question responses: 128 Blank responses: 35 (21.5%)	14 (8.5%)	15 (9.2%)	23 (14.1%)	24 (14.7%)	42 (25.8%)	10 (6.1%)	
22. I think good treatments are available on the NHS if women experience problems with their sex lives. Total question responses: 146 Blank responses: 17 (10.4%)	11 (6.7%)	15 (9.2%)	13 (8.0%)	24 (14.7%)	44 (27.0%)	39 (24.0%)	
23. It is normal for women to have less enjoyable sex lives as they get older. Total question responses: 135 Blank responses: 28 (17.2%)	29 (17.8%)	39 (24.0%)	17 (10.4%)	16 (9.8%)	17 (10.4%)	17 (10.4%)	

**Table 3:** Identification of seven vulval body parts. Participants were shown a labelled line-drawing of the vulva and were asked, “Give the name of the body part, or describe what it does, or leave the answer blank if you do not know the name or what it does.”.

Correct Anatomical Name of Body Part (Diagram Letter)	Number of Blank Responses (Percentage of 2A Participants [n = 77 (100%)])	“Do Not Know” Typed as a Response (Percentage of 2A Participants [n = 77 (100%)])	Correct Response Number of Correct Responses (Percentage of 2A Participants [n = 77 (100%)]) [Correct Answer Groupings (Number of Codes per Group)]	Mis-Identifications Number of Mis-Identifications (Percentage of 2A Participants [n = 77 (100%)]) [Mis-Identification Group (Number of Codes per Group)]
Labia majora (A)	30 (39.0%)	3 (3.9%)	44 (57.1%) [Labia majora (13), Vulva (7), Outer lips (6), Other words describing lips (18)]	0
Labia Minora (B)	38 (49.4%)	4 (5.1%)	34 (44.2%) [Labia minora (12), vulva (2), Other words describing lips (19)]	1 (1.2%) [Vagina (1)]
Clitoris (C)	27 (35.1%)	4 (5.2%)	39 (50.6%) [Clitoris (35) Clit (3) Clitoral hood (1)]	7 (9%) [Vulva (2), pee hole, urethra, small labia, urinary opening, labia majora]
Urethra (D)	30 (40.0%)	5 (6.5%)	22 (28.6%) [Urethra (18), pee hole (3), urethral opening (1)]	20 (26.0%) [Vagina (1) Clitoris (19)]
Vagina (E)	26 (33.8%)	3 (3.9%)	44 (57.1%) [vagina (41), opening (1), introitus (2)]	4 (5.2%) [Perineum (1), urethra opening (1), vulva (2)]
Perineum (F)	48 (62.3%)	7 (9.1%)	17 (22.1%) [Perineum (14), Vulva (1) Skin at back of vagina (1) Gooch (1)]	5 (6.4%) [Anus (1), vagina (1), peritoneum (1), part of vagina (1), Bartholin’s gland (1)]
Anus (G)	31(40.3%)	3 (3.9%)	44 (57.1%) [Anus (39), bottom/bum hole (5)]	2 (2.6%) [Mole (1), perineum (1)]

**Table 4:** Participant 2B responses. n = 80. A line-drawing of the vulva was shown, without a title or anatomical name, followed by the question, “What would you call the whole area of the body shown in this picture…”.

2B Question Stem: What Would You Call the Whole Area of the Body Shown in This Picture…	Main Themes Emerging, Extracted from Free-Text Responses.	Individual Typed Responses (Number of Identical Responses)
…when speaking with a romantic or sexual partner.	Vagina, No words used. Vulgar word/“swear word” Female parts/bits, “Down below”	Vagina (4) Vagina, sex (1) Vagina or downstairs (1) Vagina, vag, private part (1) I probably would use the proper words as much as I knew them (1) Not sure (1) None (2) Pussy (2) Fanny (1) Cunt (1) Lips, fanny, ladygarden (1) ‘Wee warm place’ is a personal joke between myself and husband (1) Girlie bits (1) Lady parts (1) Girl bits, “down below”, genitalia—it really depends very much on the context (1) Private parts (1) Bits (1) Last bits (1) Front bits, down below (1) Down there (1) ‘Down below’ or I wouldn’t use words, it would be uncomfortable (1)
…when speaking with your parent or carer, as a child.	Never discussed. Bottom/bum, Words only understood within the family. Private parts. Vagina/anatomical term.	None (6) I wouldn’t talk about it (1) I don’t think we had a special word (1) Never spoken about it (1) Never talked about it (1) Unmentionables (1) I have never used words (1) Didn’t (1) No (1) Not sure (1) Usually I used words from our native language which was not English, I don’t really remember using any English words, we didn’t talk about it much. (1) N/A (1) Not a subject I ever talked about with my mother, nor she with me. She gave me a book to read when I left Junior school entitled “You’re a young lady now” by Lilia White. End of sex education at age 11. (1) Bum (3) Front bottom (3) Bottom (2) Front bum (1) Privates (2) Private parts (5) My private parts or tute (1) Fanny, private part (1) Genitals (1) Groin (1) Vagina (3) V (1) Fairy (1) Twinkle (2) Lady parts (2) Nunny/front bottom (1) Loo loo (1) Noo noo (1)
…when speaking with a healthcare professional	Vagina, Genitals, Uncertain what word to use, Female parts, Private parts, Vulva, Other scientific terms.	Vagina (34) I would probably still say vagina (1) Virginia (1) Vagina, vulva, labia (1) Vagina/genitalia (3) Vulva, vagina, perineum, genitalia (1) If I knew the exact word and where the exact problem lay, I’d use it, otherwise say eg fanny lips or inside my vagina (1) Genitals (4) Genital area (1) Genitalia (1) Scientific terms (1) I would use the proper words if I knew them but for the ones I don’t I might try to descrive what I am refering to (1) I would use specific words for each body part or genitals as a general term (1) Reproductive organs Cervix (during cervical exams) (1) bum hole (1) I would use formal words depending on what part I needed to discuss eg vulva, anus etc. (1) Bum (1) Vulva (4) Not sure (1) Don’t know (1) Don’t know- I would avoid using a word and hint at it. (1) I have no idea what the collective name for these body parts would be, so I’d probably just point and look embarrassed. (1) Female parts (1) Female area (1) Privates (2) Private parts (1) I might say private area, I don’t know (1)

**Table 5:** Free-text comments: main themes and quotes to illustrate these themes.

Thematic Analysis of Free-Text Comments: Main Themes Emerging	Quotes to Illustrate Theme
Women’s blood sugar management is diabetes HCPs’ priority	“Trying to have conversations about sexual desire/dysfunction with diabetes HCPs is challenging because they really don’t appear to be comfortable discussing anything beyond HbA1c, Time in Range or lipid profiles.” “Usually my diabetes team barely gets through asking me about hypos and trying (and failing) to download my glucose readings,”
Diabetes HCPs discuss sex in connection to contraception and planning pregnancy rather than to promote positive sexual health or sexual enjoyment.	“I’ve spoken about sex connected to contraception and planning pregnancy but not about just sex for its own sake” “I have been asked about my sex life to do with avoiding getting pregnant without planning it for my diabetes but no one has spoken about my sex life and diabetes otherwise. It is hard enough starting relationships without managing diabetes as well.”
Lack of awareness of a link between diabetes and sexual problems	“I was also unaware that diabetes can affect my sex life.” “I haven’t thought about this being connected with diabetes before”
Taboo/strong expectation of silence about sex during diabetes care.	“Unless you educate yourself, no-one discusses it, it is a taboo” “Never been asked about sexual matters at diabetic reviews. My DN would fall off her chair if I brought it up.”

## Data Availability

The data presented in this study are available on request from the corresponding author due to privacy/ethical restrictions.

## Supplementary Materials

The following supporting information can be downloaded at: https://www.mdpi.com/article/10.3390/healthcare13212743/s1, Supplementary File S1: CHERRIES reporting template; Table S1: Main Survey Question Responses (Results presented in Table 2, expressed as number and percentage of respondents per question).
