# Supplementary material for: Discussing Sexual Health During Diabetes Care, a Survey of UK Women—My Diabetes Nurse “Would Fall off Her Chair If I Mentioned It”
Source: Healthcare (Basel). 2025 Oct 29;13(21):2743. doi: 10.3390/healthcare13212743 (PMC12609788; doi:10.3390/healthcare13212743)
Supplement: Supplementary file 1 [file healthcare-13-02743-s001.zip › healthcare-3892115-supplementary.pdf]

**Supplementary File S1**

Contents:

1- CHERRIES Reporting template
2- Further information on Part 2 analysis
3- Supplementary Table S1 (Part 1 results expressed as percentage of participants who selected a response)

## 1. CHERRIES Reporting Template

**Survey Title:**
**Discussing Sexual Health During Diabetes Care, a Survey of UK Women. My Diabetes Nurse "Would Fall Off Her Chair if I Mentioned it."**

### 1. Design

**Survey design**
A cross-sectional on-line survey

**Target population / sample frame**
UK-resident women, aged 16+, diagnosed with diabetes (any kind or treatment) by an HCP.
Self-identification as a woman, regardless of gender, sexuality, or sex at birth.

As an exploratory survey, there was no minimum sample number but recruitment aimed to include as representative a sample of the UK population as possible, and therefore the survey was advertised widely. Because the survey aimed to provide further information about communication and silences in the healthcare dynamic of diabetes care, following a survey of 111 HCP participants on a similar subject, recruitment aimed for a similar sample size.
A maximum of 300 participants was set, for pragmatic reasons of data analysis.

**Sampling approach**
Participation was voluntary (volunteer or non-probability convenience sampling)

**2. Ethical Approval and Informed Consent**
The study received ethical approval from researcher contact details in original document

All participants read participant information (available to view at the end of this document), had the opportunity to ask questions and provided informed consent electronically before beginning the online questionnaire.

Participant information:

**Informed consent process: The following form approximates the online form**

**INFORMED CONSENT FORM**

**Thank you for considering taking part in this research.**

**Please complete this form after you have read the Information Sheet and/or listened to an explanation about the research.**

**Title of Study:**

Talking about sex with healthcare professionals during diabetes check-ups: a survey of UK women living with diabetes

**[Researcher contact details in original document]**

**1.11.2024**

The person asking for your consent must explain the project to you before you agree to take part. If you have any questions about the Information Sheet or their explanation, please ask the researcher before you make your decision. You will be given a copy of this Consent Form and the Information Sheet to keep and refer to at any time.

You will be asked to complete this document online before participating in the research when you click on the participate/ questionnaire link.

By clicking "I agree" to statement, you are consenting to this part of the study. Any un-clicked statements will mean that you DO NOT agree to that part of the study and this may mean you are ineligible for the study.

| **Taking part in the study** | | |
|---|---|---|
| | Statement | Online, each box appears as a statement in Qualtrics with "I agree" |
| **1** | I confirm that I have read and understood the participant information sheet dated 1.11.2024 for the above study. I have had the opportunity to consider the information and ask questions which have been answered satisfactorily. | |
| **2** | I understand that my participation is voluntary and that I am free to withdraw at any time during the study without giving any reason. Furthermore, I understand that data already collected can be withdrawn up to the point I finish the online questionnaire and press the "finish and submit answers" button. If I do not press this button, all data will be deleted on 2nd February 2025. If I press this button and wish to withdraw my answers after this time, I can do this up to 1st February, 2025 by emailing the research team on j.c.murphy@surrey.ac.uk and providing my password. After 1st February, 2025 it will no longer be possible to withdraw my data. | |
| **3** | I understand that information I provide may be subject to review by responsible individuals from The University of Surrey and/or regulators for monitoring and audit purposes. | |
| **4** | I understand that information I provide will be used in various anonymised outputs, including academic published articles, reports, presentations and made available on the study website. | |
| **5** | I understand that my personal data, including this consent form, which link me to the research data, will be kept securely in accordance with data protection guidelines, and only be accessible to the immediate research team or responsible persons at the University. | |
| **6** | I understand any personal contact details collected about me, such as my email address, if I chose to provide it for the purpose of being contacted about future studies, will not be shared beyond the study team. | |

| | | |
|---|---|---|
| **7** | I consent to the processing of my special category data (including race and ethnic origin, religion and any details of religious or philosophical beliefs I mention in my responses) for the purposes stated in the information sheet. | |
| **8** | I agree to take part in this study. | |

| **Signatures** | | |
|---|---|---|
| **Your own name is not recorded as this is an anonymous study, but you must click to agree to all the above statements to participate** | | |
| **Researcher name** | **Researcher Signature** | **Date** |

**Data protection / privacy measures**

Data was gathered using Qualtrics. This included research data, personal data (age and email address, where provided), types of special category data (religion, ethnic origin, health and sex life data).

Collected data is accessed via a password protected laptop. Data was analysed using university software including SPSS/ Excel and stored on the university Onedrive in

accordance with University policy. Before transfer to SPSS/ Excel, identifying data will

be removed and data anonymised.

If data is to be shared with the external supervisor outside the research team at [contact details in original document] anonymised data only will be shared and personal data will not be shared.
If participants chose to provide an email address to participate in the prize draw, this was stored in the secure drive until the end of data collection, used only for the purpose of selecting a random prize draw winner and contacting them and then destroyed. If participants choose to provide data in order to be informed about future studies they may wish to participate in, the addresses were be kept in the secure drive, accessible only to Joanna Murphy and deleted at the end of the research series or in 2029, whenever is sooner. Once data has been transferred to the secure drive, it was deleted from Qualtrics.

Care is taken with the storage of the laptop and with passwords.

## 3. Development and Pre-Testing

### Survey development process

The survey was conducted after an extensive literature review which identified possible barriers to discussion between healthcare professionals HCPs and women with diabetes regarding sexual health. A PPI discussion regarding the survey themes also took place between a researcher and 3 women with diabetes before survey design.

This is an under-researched area and the survey is exploratory. No valid survey tools exist to study communication dynamics between women and HCPs regarding sexual health. Part 1 of the survey tool was designed in an iterative process after a previous HCP survey:

Murphy, J. C., Cooke, D., Griffiths, D., Setty, E., & Winkley-Bryant, K. (2024). Asking women with diabetes about sexual problems: An exploratory study of NHS professionals' attitudes and practice: A survey of healthcare professionals regarding communication and silences about sexual problems during the routine care of women with diabetes. *Diabetic Medicine*, *41*(8), e15370.

This study had included several stages in the validation process as described below as described below (cited from the research article):

"No existing validated questionnaire for studying communication in this population was identified, so steps were followed to develop (and begin to validate) the survey during the pilot process, following the methodology of Boateng et al.[19] Exploratory questions were

developed following a literature review and piloted using a ‘think-aloud’ technique.[20] The survey proforma was completed by 10 pilot participants, all HCPs working regularly with women with diabetes, who were audio recorded whilst thinking aloud during the task. Transcripts were analysed for opportunities to improve phase 2 data collection.”

Like the HCP survey, in the current survey, women are asked to provide demographic information and to select from Likert categories or narrative response options to provide information on their experience, expectations and concerns regarding discussing sexual health with HCPs during diabetes care. Where appropriate, question stems covered the same themes presented to HCPs, asking about belief in availability of effective treatment, expectations women’s sexual enjoyment as women age, awareness of link between diabetes and high risk of sexual problems for women, whom women would approach first to discuss sexual problems, preference for gender of HCP to discuss the topic, expectations of discussing the topic (surprise, annoyance/ offence but also including positive responses such as being pleased or discussing a previously undisclosed problem). In addition, a scenario is included regarding HCP gender, to attempt to ask women to consider professionals where the only difference is apparent gender rather than age or other visible attribute, nor clinical skills. The questions also include women’s expectations of HCP responses, mirroring the HCP survey which asked about HCP expectations of women.

The survey also included an optional second part (parts 2A and 2B). Part 2A closely followed the structure of a previously published but unvalidated tool:

El-Hamamsy, D., Parmar, C., Shoop-Worrall, S., & Reid, F. M. (2022). Public understanding of female genital anatomy and pelvic organ prolapse (POP); a questionnaire-based pilot study. *International urogynecology journal*, *33*(2), 309-318.
Part 2B posed a further question in 3 parts, and is exploratory, with wording developed de novo following literature review.

All parts of the survey were tested by 5 pilot participants, meeting eligibility criteria. Pilot participants commented favourably on clarity and ease of completion of the survey and no need for change was identified.

After the first 3 participants attempted to complete part 2A, the research team was contacted by participants to say participants could not enter 2A results (though other results were unaffected). This was due to a programming error which had been introduced during the process of making the survey live, after the pilot study, and was corrected for subsequent participants.

**Usability / technical testing**
The survey tool followed development of a survey tool for HCPs, and navigation followed a similar logic.

The survey was tested by the research team and by pilot participants for functionality on mobile phone and computer.

## 4. Recruitment / Administration

**Open vs. closed survey**

Recruitment to the survey was open, though this included contacting HCPs via professional networks who could invite participants if they wished to. Any such invitation was an open invitation.

**Contact mode / Initial contact/ Advertisement / Announcement of survey**

The study was advertised on the Diabetes UK website, social media, Facebook, and Twitter pages aimed at women with diabetes (all types). Posts appeared in Facebook groups for Black British, Muslim, and Indian women.
The survey was promoted to diabetes HCPs through informal professional networks and social media, including HCP-focused Facebook groups, Twitter, and LinkedIn.
The number and demographic information of potential participants who read invitations to participate but chose not to take part could not be recorded, because of the nature of the open invitation and voluntary participation.

Recruitment wording:

Are you interested in taking part in diabetes research?
Researchers at The University of Surrey are looking for participants for an online survey about discussing sexual health at diabetes appointments:
We are inviting:

- Women (aged 16 and over)
- Living in the UK
- Who have been diagnosed with diabetes of any kind (and on any treatment)

The survey asks about women's experience and attitudes regarding discussing sexual health (sex life) with healthcare professionals.
It takes about 15 minutes to complete online.
It is anonymous and voluntary.
You can take part anytime from now until 01.02.2025
Participants who complete the survey are eligible to enter a £100 Amazon voucher prize draw.
Please click this link [Qualtrics link to participant information] for more information or contact the research team j.c.murphy@surrey.ac.uk

This study was reviewed and given a favourable ethical opinion by The University of Surrey Ethics Committee (**FASS 23-24 067 EGA)**

**Voluntary / mandatory participation**
Participation was voluntary

**Incentives**
No incentives were offered but participants who submitted a completed survey were eligible for entry in a prize draw with a single £100 voucher prize.

**Time period of data collection**
1st November 2024- 1st February 2025

**5. Survey Implementation Details**

**Randomization of item order / questionnaire versions**
Items were not randomized.

**Adaptive questioning / branching logic**
No adaptive questioning nor branching logic was used.

**Number of items per page**

1- 4 items per page:

**Number of pages / screens**
3 pages: introduction and informed consent (not including downloads for participant information and consent form)

6 pages to confirm eligibility to participate (1 question per page) followed by 1 page asking the participant to make up a password in case they wish to withdraw data before the date of data pooling and analysis.

3 pages: questions regarding demographic details

17 question pages with Likert and narrative response options (thirteen pages with 1 question, 3 pages with 2 questions, one page with 4 questions: questions shown in the results tables of the survey).

End of part 1: 1 page stating:

The survey is almost finished. There are 5 more questions in this survey.
These last questions are about how much women know about the sexual or private parts of their body, and words they would use to talk about them.

The questions include a drawing of women's private (sexual) parts.

We are asking these questions because we want to understand more about how women understand and talk about the sexual or private parts of their bodies.

You can skip these questions if you wish and finish now (you can enter the prize draw if you submit answers now or at the end of the last questions).

If participants wish to finish, they are taken to the debrief pages.

Part 2:

3 pages with labeled diagrams and free text response options.

3 pages with 3 free text response options.

1 page with an overall free text “other comments” invitation.

4 pages debrief information.

Survey questions are shown on the results tables. The full survey instrument is available on request.

**Completeness / consistency checks**

The online survey programming automatically performed completeness checks to ensure that all mandatory questions were answered and only eligible participants, who had provided informed consent to participate could proceed to data collection and submission. Respondents were prompted to complete missing items if they attempted to proceed without answering required fields.
For non-mandatory questions (once consent and eligibility questions were answered) participants could skip one or more answers and leave responses blank. Researchers manually checked answers for straight lining, on the Excel and SPSS spreadsheets, though this was not detected.

Item level missingness or “blank responses” is presented in the results. For Part 1 this varies from a minimum of 6/163 (3.7% Wilson CI 1.7- 7.8%) for Question 3 to a

maximum of 54/163 (33.1% Wilson CI 26.3%–40.6%), Question 20. Because all Part 1 participants viewed each question, we chose to treat each question as having 163 participants, including blank responses in the analysis alongside responses where a response category is selected. In this dynamic interested in silences, we consider silent responses to be important. This was also the approach to analysis of item level missingness in the exploratory HCP survey which informed the development of the current research. However, Supplementary table 1 shows a further analysis, with Part 1 percentages calculated from total answers to the individual question, rather than 163 participants who viewed the question.

No manual data cleaning was necessary after survey closure.

**Review step / editing responses**
Respondents could review and edit responses before submission and were advised the survey was about to end and to make any changes before submission.

**6. Handling Multiple Entries & Unique Visitors**

**Definition of a unique visitor**
The survey tool used a cookie to identify visitors who had previously begun to participate in the survey, if they used the same device to access the questionnaire. This allowed them to pick up where they left off. In addition, researchers manually checked the IP addresses to ensure these were unique numbers.

**7. Analysis**

**Handling of incomplete / partial responses**
Data was only analysed when a participant finished the survey and clicked to submit their data. This could take place either at the end of Part 1 or at the end of Part 2. Data from incomplete responses was discarded at the end of the data collection period.

We did not exclude participants based on timestamp or survey completion speed.

We did not apply weighting or statistical correction.

**8. Response Rates & Metrics**

**View rate**
As an anonymous study advertised on social media we are unable to assess view rate. Some of the larger social media sites have many thousands of followers (for example Diabetes UK Facebook has 315, 000 followers on the main page) but posts advertising

academic research invitations are not advertised prominently within these large sites, and may only be visible for brief periods of time, and not viewed by all followers.

**Participation rate**
Since this is an anonymous, voluntary study and we are unable to assess view rate, we cannot assess participation rate

**Completion rate**
198 people read the participant information and gave informed consent to participate. 163 people (82.3%) completed the survey (Part 1) and submitted answers.

80 people completed part 2 of the survey (40.4%). NB 3 participants had their data excluded from part 2A because of a programming error affecting their ability to enter part 2A responses but continued to Part 2. All 80 participants who began Part 2 completed it (100%).

**Completeness rate**
Blank responses to individual questions are presented in the results page.

**Appendices: Participant Information**

Participant information (this was available to participants online)

**<u>Invitation Paragraph</u>**

We would like to invite you to participate in this University of Surrey research project.

The study forms part of Dr Joanna Murphy's PhD research project.

You should only participate if you want to; choosing not to take part will not disadvantage you in any way.

Before you decide whether you want to take part, it is important for you to understand why the research is being done and what your participation will involve. If you have any questions, you can contact us using the contact details at the end of this information sheet.

**What is the purpose of the study?**

This study gathers current data from women, aged 16 years or older, with a diagnosis of diabetes.

Recent research shows women with diabetes are at high risk of sexual problems, but they often do not speak to professionals about them, and professionals usually do not ask about them.

The survey will ask about women's experience, unmet needs, and attitudes to being asked about sexual health at diabetes appointments. It will collect data about the backgrounds of the women competing the survey to assess whether this is a diverse group.

We will undertake further research, guided by the findings of this study and will interview women about this subject. We will offer participants the option to be invited to participate in future research on this topic by supplying their email address.

**Who is responsible for this study?**

This study is the responsibility of Dr Joanna Murphy at The University of Surrey.

**Why have I been invited to take part?**

You are invited to participate in this study because you are a:

A woman, and

aged 16 years old or older, and

diagnosed by a healthcare professional with diabetes, and

you live in the UK

We invite all people who self-identify as a woman to participate.

Women with diabetes of any kind are invited to participate, regardless of how long ago it was diagnosed and what treatment is used.

Women who currently live in the UK (England, Northern Ireland, Scotland and Wales) are invited to participate.

If unsure, please email the research team on j.c.murphy@surrey.ac.uk prior to participating.

We have advertised this study through Diabetes UK, and publicised it through social media (Facebook, X, Instagram). We have also asked healthcare professionals to share links with their patients, and spoken to professionals' groups, asking them to tell their members about the study.

**Do I have to take part?**

Participation is voluntary and you do not have to take part.

We will describe the study in this information sheet. You can read this information sheet for as long as you wish before deciding whether you wish to take part, so long as the survey is completed by

Please contact us if there is anything that is not clear, or if you have any questions, or need more information.

**What will happen to me if I decide to take part?**

If you decide to take part, you will be given this information sheet to keep. You then click on the link below to access the anonymous online survey:

When you click on the link, you will first be asked to sign a consent form to confirm your agreement to participate.

The survey is totally anonymous. We ask you to make up a 5-digit password in case you contact us later about your answers.

We ask everyone questions at the start of the survey to check they are eligible to participate. After these questions are answered, you are shown the survey questions. You are free to answer any question or not answer any question, or to end the questionnaire completion at any stage.

The survey takes about 15-20 minutes to complete online.

You will be able to download and keep a copy of this information sheet and consent form.

If you temporarily need to leave the survey before finishing it, you can restart where you left off by using the same link on the same device. If you use a different device, the survey will restart.

**<u>What happens if I do not want to take part or if I change my mind?</u>**

You are free to decide not to take part, or to withdraw from the study at any time until you complete the questionnaire by pressing the "finish" button, without giving a reason.

**<u>What happens to my data if I want to withdraw?</u>**

If you wish to remove or change your survey answers, you may do this any time till you press the "finish survey and submit answers" button on the questionnaire.

No answers will be used by the research team until you press the "finish survey or submit answers" button.

You may choose not to answer any single question or all questions.

If you begin the survey but wish to withdraw before finishing, please do not press the “finish survey and submit answers” button. No data from your questionnaire answers will be used by researchers unless you finish the survey by pressing “finish survey and submit answers” button.

Incomplete data will be deleted on 02 February 2025

If you finish the questionnaire and press the “finish” button, data from your answers will be visible to researchers. On 1st February 2025 we will finish collecting survey data and close the survey for everyone. All answers will anonymised and pooled on this date. You will not be able to withdraw your data after this time.

Before 1st February 2025 if you have submitted answers but want change your mind and to withdraw your data (remove your answers from the study), you can contact the researchers, giving your 5 digit password. Researchers will try to identify and remove your data. After 1st February (from 2nd February 2025 onwards) you cannot remove your data.

At the end of the survey, we will ask whether you wish to provide the research team with your email address, to participate in a prize draw, or to be invited to participate in future studies, if relevant.

If you chose to provide your email address, your email address will only be used to invite you to participate in future studies about sexual health and diabetes, and/ or to participate in the prize draw.

Your email will be kept until the completion of all the series of studies in the postgraduate project (a maximum of seven years) and will be used only to invite you to participate in future research on similar topics, if relevant. We will send a maximum of 2 email invitations about each new study.

Your email address will not be used for other reasons nor distributed to people outside the research team. We will delete your email address immediately if you email j.c.murphy@surrey.ac.uk

## What are the possible benefits of taking part?

There are no direct benefits to you from taking part. However, we hope the information will help improve care in general for women with diabetes. Depending on findings, the researchers will discuss this research at conferences, in professional journals, via voluntary agencies such as Diabetes UK, and to others as needed.

## Are there any potential risks involved?

A possible disadvantage to taking part in the study is time to complete the study. Sexual problems can also be upsetting to discuss or think about.

Please only participate if comfortable to do so. If support is needed for distress caused by the issues raised in the study, please see the support section below.

Unfortunately, sexual violence and abuse is not uncommon. Researchers are not able to identify participants or to respond to disclosures regarding sexual violence made in free text comments. Resources regarding support for women who have experienced sexual

violence or sexual abuse are below and participants are encouraged to contact these directly.

**How is the project being funded?**

This research is funded as a postgraduate research project with University of Surrey.
The prize draw prize is funded by the research budget of Dr Kirsty Winkley

**Will my participation be kept confidential?**

We are responsible for making sure your participation is kept confidential and any data is kept secure and used only in the way described in this information sheet.

Your information may be reviewed for monitoring and audit purposes by University of Surrey and/or regulators who will treat your data in confidence.

Your participation is totally anonymous, and you will not be identifiable from your survey entry in any publication or report. If you mention any potentially identifiable data, we will anonymise it in any report or publication.

## Will my data be shared or used in future research studies?

We would like your permission to share the anonymised data you provide us, publicly, in professional journals, reports and presentations.

The data will also be used as part of a PhD thesis.

Your email address, if supplied, will only be used for the purpose of inviting you to participate in future research and will not be shared, linked to your survey answers or pilot study answers, nor used in any other way

We will not share your data with any other party

## What will happen to the results of the study?

This research may be published in academic journals, professional reports, and part of a PhD thesis

You can contact the study team to find out the results of the research, which is likely to be available in April 2025.

**Who has reviewed this study?**

This research has been reviewed by an independent group of people, called an Ethics Committee. This study was reviewed and given a favourable ethical opinion (**FASS 23-24 067 EGA)**

## Section: Your personal data

**What is personal data?**

‘Personal Data’ means any information that identifies you as an individual. We will be collecting and using some of your personal data that is relevant to completing the study and this section describes what that means.

The information that we will collect may include your email address, if you choose to provide this, which is regarded as ‘personal data’ and religion and ethnic origin, which is regarded as a ‘special category personal data’. We will use this information as explained in the ‘What is the purpose of the study’ section above.

**Who is handling my personal data?**

Dr Joanna Murphy (j.c.murphy@surrey.ac.uk) at The University of Surrey has the legal responsibility for managing the personal data in this study, will act as the 'Data Controller' for this study. The research team will process your personal data on behalf of the controller and is responsible for looking after your information and using it properly.

**What will happen to my personal data?**

As a publicly funded organisation, we must only use **identifiable personal** information from people who have agreed to take part in research and process this data fairly and lawfully. The University of Surrey
processes personal data for the purposes of carrying out research in the **public interest** and special category data is processed on an additional condition necessary for **research purposes.** This means that when you agree to take part in this research study, we will use and look after your data in the ways needed to achieve the outcomes of the study.

Your personal data will be held and processed in the strictest confidence, and in accordance with current data protection regulations. When acting as the data controller, the University will keep identifiable information about you for 7 years after the study has finished after which time any identifiers will be removed from the aggregated research data.

Your rights to access, change or move your information are limited, as we need to manage your information in specific ways or the research to be reliable and accurate. If you decide to withdraw from the study, we may not be able to withdraw your data. We will keep and use the minimum amount of your personally identifiable information that we have already collected in order to complete the study.

If you wish to complain about how we have handled your personal data, you can contact our Data Protection Officer who will investigate the matter (contact details in original document)

If you are not satisfied with our response or believe we are processing your personal data in a way that is not lawful, you can contact the Information Commissioner's Office (ICO) (https://ico.org.uk/).

You can find out more about how we use your information by contacting j.c.murphy@surrey.ac.uk

## Section: Further information

**What if you have a query or something goes wrong?**

If you are unsure about something, you can contact the research team for further advice using the contact details at the bottom of this information sheet.

However, if your query has not been handled to your satisfaction, or if you are unhappy and wish to make a formal complaint to someone independent of the research team, then please contact:

(contact details in original document)

The University has in place the relevant insurance policies which apply to this study. If you wish to complain or have concerns about how you have been treated during the course of this study, then you should follow the instructions given above.

## Who should I contact for further information?

Dr Joanna Murphy

J.c.murphy@surrey.ac.uk

You can also speak to the project supervisor:

[contact details available on the original form]

If you have any questions or require more information about this study, please contact the research team using the following contact details:

(address contact details in original document)

## Sources of support

If you are affected or distressed by the subject of this research and would like information or support, please contact the following organisations:

**Emotional support:**

If you are in emotional distress and need to speak to someone urgently, call your local crisis line (accessible via 111) or in an emergency attend A&E or call 999.

Samaritans is a charity, which offers a listening telephone service 24/7 on 116 123

**Resources about diabetes and sexual health:**

**Your GP can provide information about diabetes and sexual health.**

You can also make an appointment at a sexual health clinic and can contact 111 for your nearest centre. You can also find your nearest clinic by entering your postcode on this website: https://www.nhs.uk/service-search/sexual-health/find-a-sexual-health-clinic/

**National Sexual Health Helpline**
If you would like to talk to someone about sexual health you can call the national sexual health helpline free on **0300 123 7123**. Your call will be treated with sensitivity and in the strictest confidence. The Sexual Health helpline is open from 9am -
8pm Monday to Friday.

Information about speaking to a partner about painful sex

https://patient.info/news-and-features/how-to-talk-to-your-partner-about-painful-sex

Patient information about diabetes and sexual health available from Diabetes UK:

https://www.diabetes.org.uk/guide-to-diabetes/complications/sexual-problems-women

Information about women's sexual problems from the NHS: https://www.nhs.uk/live-well/sexual-health/female-sexual-problems/

Support following sexual violence:

Please do not disclose sexual violence or abuse on the survey as we do not have your contact details to offer help. Instead, please access services below, if you need help following sexual violence.

Where someone could currently be at risk from sexual violence, please contact the police (999 in an emergency)

Rape Crisis offers support and help after rape, sexual assault, sexual abuse or any form of sexual violence: Helpline 0808 5002222, www.rapecrisis.org.uk

The Survivors' Trust can signpost you to specialist rape and sexual abuse support in your area: www.survivorstrust.org (you can enter your postcode to find a list of services in your area)

Victim Support can offer help after crime. Their support line is 08081689111

**Thank you for reading this information sheet and for considering taking part in this research.**

## 2. Further information on Part 2 analysis and coding information

Coding Matrix 2A

Initial phases involved 2 researchers individually familiarising themselves with the data. Entries were single response (one or two words) and each entry was entered into one grouping only. Both researchers met to compare groupings, which were extremely similar and required only decisions about naming and infrequent decisions regarding inclusion into groups. The coding matrix below shows the decisions made after reaching consensus.

Body Part A: Labia majora

| | Emergent groups | | | | |
|---|---|---|---|---|---|
| Individual responses | Labia majora including phonetic spelling | Outer lips | Other words describing labia/ lips | Vulva | Judged correct by both researchers after discussion and consensus |
| Labia majora | 1 | 0 | 0 | 0 | 1 |
| Labia majora | 1 | 0 | 0 | 0 | 1 |
| Vulva | 0 | 0 | 0 | 1 | 1 |
| Outer lips | 0 | 1 | 0 | 0 | 1 |
| Labia | 0 | 0 | 1 | 0 | 1 |
| Labia major | 1 | 0 | 0 | 0 | 1 |

| | | | | | |
|---|---|---|---|---|---|
| Outer labia | 0 | 1 | 0 | 0 | 1 |
| Labia majora | 1 | 0 | 0 | 0 | 1 |
| Labia | 0 | 0 | 1 | 0 | 1 |
| Lips | 0 | 0 | 1 | 0 | 1 |
| Outer lips | 0 | 1 | 0 | 0 | 1 |
| Labia majoris | 1 | 0 | 0 | 0 | 1 |
| Lips | 0 | 0 | 1 | 0 | 1 |
| Vulva | 0 | 0 | 0 | 1 | 1 |
| Labia majora | 1 | 0 | 0 | 0 | 1 |
| Labia majora | 1 | 0 | 0 | 0 | 1 |
| Labia | 0 | 0 | 1 | 0 | 1 |
| Lips | 0 | 0 | 1 | 0 | 1 |
| Vulva | 0 | 0 | 0 | 1 | 1 |
| Outer lips | 0 | 1 | 0 | 0 | 1 |
| Vulva | 0 | 0 | 0 | 1 | 1 |
| Outer labia | 0 | 1 | 0 | 0 | 1 |
| Labia majora | 1 | 0 | 0 | 0 | 1 |
| Vulva | 0 | 0 | 0 | 1 | 1 |
| Labia | 0 | 0 | 1 | 0 | 1 |
| Vigina lip | 0 | 0 | 1 | 0 | 1 |
| Labia majora | 1 | 0 | 0 | 0 | 1 |
| Labia majora | 1 | 0 | 0 | 0 | 1 |
| Lips | 0 | 0 | 1 | 0 | 1 |
| Lips | 0 | 0 | 1 | 0 | 1 |
| Labia | 0 | 0 | 1 | 0 | 1 |
| Labia | 0 | 0 | 1 | 0 | 1 |
| Labia magora | 1 | 0 | 0 | 0 | 1 |
| Lips | 0 | 0 | 1 | 0 | 1 |
| Outer labia | 0 | 1 | 0 | 0 | 1 |
| Labia magoria | 1 | 0 | 0 | 0 | 1 |
| Vulva | 0 | 0 | 0 | 1 | 1 |
| Vulva | 0 | 0 | 0 | 1 | 1 |
| Labia | 0 | 0 | 1 | 0 | 1 |
| Lips | 0 | 0 | 1 | 0 | 1 |
| Venus lips | 0 | 0 | 1 | 0 | 1 |
| Big lips | 0 | 0 | 1 | 0 | 1 |
| Labia | 0 | 0 | 1 | 0 | 1 |
| Laba majoa | 1 | 0 | 0 | 0 | 1 |
| Dk | | | | | 0 |

| | | | | | |
|---|---|---|---|---|---|
| Dk | | | | | 0 |
| Dk | | | | | 0 |

Initially researcher 1 (marginally) judged responses with vulva to be incorrect, but noted this as a discussion point due to uncertainty in this allocation, whereas researcher 2 judged them to be correct. After discussion both researchers agreed to judge them correct as per methodology.

Discussion occurred regarding whether to make a "DK" subgroup but decision made to report these separately from the blank responses but not to form a theme.

Discussion occured about grouping labia majora and labia, or lips and labia, before finalising groups.

Body Part B: Labia minora

| Individual responses | Emergent groups | | | | Judged Correct By both researchers |
|---|---|---|---|---|---|
| | Labia minora including phonetic spelling | Other answer including Lips/ labia | Vulva | vagina | |
| Lip | 0 | 1 | 0 | | 1 |
| Labia minora | 1 | | 0 | | 1 |
| Labia | 0 | 1 | 0 | | 1 |
| Labia minora | 1 | | 0 | | 1 |
| Labia minora | 1 | | 0 | | 1 |
| Labia minoria | 1 | | 0 | | 1 |
| Inner labia | 0 | 1 | 0 | | 1 |
| Inner lips | 0 | 1 | 0 | | 1 |
| Vagina | 0 | | 0 | 1 | 0 |
| Labia | 0 | 1 | 0 | | 1 |
| Labia | 0 | 1 | 0 | | 1 |

| | | | | | |
|---|---|---|---|---|---|
| Lips | 0 | 1 | 0 | | 1 |
| Inner lips | 0 | 1 | 0 | | 1 |
| Vulva | 0 | | 1 | | 1* |
| Labia | 0 | 1 | 0 | | 1 |
| Inner labia | 0 | 1 | 0 | | 1 |
| Inner lips | 0 | 1 | 0 | | 1 |
| Labia minora | 1 | | 0 | | 1 |
| Labia minor | 1 | | 0 | | 1 |
| Labia | 0 | 1 | 0 | | 1 |
| Labia minoris | 1 | | 0 | | 1 |
| Labia | 0 | 1 | 0 | | 1 |
| Inside lips | 0 | 1 | 0 | | 1 |
| Labia | 0 | 1 | 0 | | 1 |
| Labia | 0 | 1 | 0 | | 1 |
| Labia | 0 | 1 | 0 | | 1 |
| Labia minora | 1 | | 0 | | 1 |
| Labia | 0 | 1 | 0 | | 1 |
| Labia menoa | 1 | | 0 | | 1 |
| Labia minora | 1 | | 0 | | 1 |
| Labia minora | 1 | | 0 | | 1 |
| Labia minora | 1 | | 0 | | 1 |
| Vulva | 0 | | 1 | | 1* |
| Lips | 0 | 1 | 0 | | 1 |
| Dk | | | | | |
| Not sure | | | | | |
| Don't know | | | | | |
| Dk | | | | | |

*Initially researcher A judged responses with "vulva" to be incorrect but noted this as a discussion point as this was a marginal decision, whereas researcher B had judged these responses to be correct. After discussion both researchers reached consensus and agreed to judge these answers as correct, as per methodology.

Discussion regarding whether to make a "DK" subgroup but decision made to report these separately from the blank responses but not to form a theme.

Discussion about separating labia from lips subgroup or grouping labia minora and labia.

Body Part C: Clitoris

| Individual responses | Emergent grouping | | | | | Judged correct by both researchers |
|---|---|---|---|---|---|---|
| | Clitoris or phonetic spelling | Clit | hood | Urinary tract structure | Labia or vulva | |
| Clitoris | 1 | | | | | 1 |
| Clitoris | 1 | | | | | 1 |
| Clitorise | 1 | | | | | 1 |
| Clitoris | 1 | | | | | 1 |
| Clitoris | 1 | | | | | 1 |
| Labia majora | | | | | 1 | 0 |
| Clitoris | 1 | | | | | 1 |
| Clitoris | 1 | | | | | 1 |
| Clitoris | 1 | | | | | 1 |
| Clitoris | 1 | | | | | 1 |
| Clitoris | 1 | | | | | 1 |
| Cliteoris | 1 | | | | | 1 |
| clitoris | 1 | | | | | 1 |
| clitersis | 1 | | | | | 1 |
| Clitoris? | 1 | | | | | 1 |
| Clitoris | 1 | | | | | 1 |
| Vulva | | | | | 1 | 0 |
| Clitoris | 1 | | | | | 1 |
| Clitoris | 1 | | | | | 1 |
| cliteris | 1 | | | | | 1 |
| Cliteral hood | | | 1 | | | 1 |
| cliteris | 1 | | | | | 1 |
| Clitoris | 1 | | | | | 1 |
| Urinary opening | | | | 1 | | 0 |
| Cliteris | 1 | | | | | 1 |
| urethra | | | | 1 | | 0 |
| Pee hole | | | | 1 | | 0 |

| | | | | | | |
|---|---|---|---|---|---|---|
| Clit | | 1 | | | | 1 |
| Small labia | | | | | 1 | 0 |
| vulva | | | | | 1 | 0 |
| Cliterus | 1 | | | | | 1 |
| Cliteris | 1 | | | | | 1 |
| Clitoris | 1 | | | | | 1 |
| Clitoris | 1 | | | | | 1 |
| cliterus | 1 | | | | | 1 |
| Clit | | 1 | | | | 1 |
| Cliterus | 1 | | | | | 1 |
| Cliterous | 1 | | | | | 1 |
| Clit | | 1 | | | | 1 |
| Clitoris | 1 | | | | | 1 |
| Clitoris | 1 | | | | | 1 |
| Cliteros | 1 | | | | | 1 |
| Clieris | 1 | | | | | 1 |
| Clitors | 1 | | | | | 1 |
| clitieros | 1 | | | | | 1 |
| Clitoris | 1 | | | | | 1 |
| Dk | | | | | | |
| Don't know | | | | | | |
| Unsure | | | | | | |
| Don't know | | | | | | |

Although both researchers judged "vulva" to be an incorrect answer, this was the subject of specific discussion after independent provisional groupings were made, and decision made to continue to consider this an incorrect response.

Discussion regarding whether to make a "DK" subgroup, but decision made to report these separately from the blank responses but not to form a theme.

Body Part D Urethra:

| Individual responses | Emergent groups | | | | Judged Correct by both researchers |
|---|---|---|---|---|---|
| | urethra | Urethral opening | Pee hole | Other | |
| Clitoris | | | | 1 | 0 |
| Urethra | 1 | | | | 1 |
| Urethra | 1 | | | | 1 |
| urethra | 1 | | | | 1 |
| Pee hole | | | 1 | | 1 |
| Urethra | 1 | | | | 1 |
| Ureathra | 1 | | | | 1 |
| Urethra | 1 | | | | 1 |
| Uretra | 1 | | | | 1 |
| Pee hole | | | 1 | | 1 |
| Or is the clit? | | | | 1 | 0 |
| Urethra | 1 | | | | 1 |
| Urethral opening | | 1 | | | 1 |
| Clitoris | | | | 1 | 0 |
| Urethra | 1 | | | | 1 |
| Pee hole 😆 | | | 1 | | 1 |
| Clitoeis | | | | 1 | 0 |
| Clitoris | | | | 1 | 0 |
| Clitoris | | | | 1 | 0 |
| Urethra | 1 | | | | 1 |
| Clitoris | | | | 1 | 0 |
| Urethra | 1 | | | | 1 |
| Urethra | 1 | | | | 1 |
| Clitoris | | | | 1 | 0 |
| Clitoris | | | | 1 | 0 |
| Urethra | 1 | | | | 1 |
| Urethra | 1 | | | | 1 |
| Clitoris | | | | 1 | 0 |
| Urethra | 1 | | | | 1 |
| Cliteris | | | | 1 | 0 |
| Urethra | 1 | | | | 1 |
| Urethra | 1 | | | | 1 |
| Clitoris | | | | 1 | 0 |
| Clitoris | | | | 1 | 0 |
| Clitoris | | | | 1 | 0 |

| | | | | | |
|---|---|---|---|---|---|
| Urethra | 1 | | | | 1 |
| Vagina | | | | 1 | 0 |
| Urethra | 1 | | | | 1 |
| Clitoris | | | | 1 | 0 |
| cliteris | | | | 1 | 0 |
| Clitoris | | | | 1 | 0 |
| cliteris | | | | 1 | 0 |
| Dk | | | | | |
| Not sure | | | | | |
| Don't know | | | | | |
| Not sure | | | | | |
| Dk | | | | | |

Researchers considered grouping urethral opening into "urethra" but decided to keep it as a separate group.

Discussion regarding whether to make a "DK" subgroup, but decision made to report these separately from the blank responses but not to form a theme.

Body Part E: Vagina

| Individual responses | Emergent grouping | | | | Judged correct by both researchers |
|---|---|---|---|---|---|
| | Vagina or phonetic spelling | opening | introitus | other | |
| Vagina | 1 | | | | 1 |
| Perineum | | | | 1 | 0 |
| Vahina | 1 | | | | 1 |
| Virgina | 1 | | | | 1 |
| Vagina | 1 | | | | 1 |
| Vagina | 1 | | | | 1 |
| Vagina | 1 | | | | 1 |
| Vagina | 1 | | | | 1 |
| Urethral opening | | | | 1 | 0 |
| Vagia | 1 | | | | 1 |
| Virgina | 1 | | | | 1 |
| Vagina | 1 | | | | 1 |
| Vagina | 1 | | | | 1 |
| Vagina | 1 | | | | 1 |
| Vagina | 1 | | | | 1 |

| | | | | | |
|---|---|---|---|---|---|
| Virgina | 1 | | | | 1 |
| Vagina | 1 | | | | 1 |
| Vagina | 1 | | | | 1 |
| Vagina | 1 | | | | 1 |
| Vagina | 1 | | | | 1 |
| Vagina | 1 | | | | 1 |
| Vagina opening | 1 | | | | 1 |
| vulva | 1 | | | | 0 |
| Opening | | 1 | | | 1 |
| Vaginal introitus | | | 1 | | 1 |
| Vagina | 1 | | | | 1 |
| Vagina | 1 | | | | 1 |
| Vagina | 1 | | | | 1 |
| Vagina | 1 | | | | 1 |
| Vagina | 1 | | | | 1 |
| Vulva | | | | | 0 |
| Vagina | 1 | | | | 1 |
| Vagina | 1 | | | | 1 |
| Vagina | 1 | | | | 1 |
| Vagina | 1 | | | | 1 |
| Vagina | 1 | | | | 1 |
| Vagina | 1 | | | | 1 |
| Vagina | 1 | | | | 1 |
| introitus | | | 1 | | 1 |
| Vagina | 1 | | | | 1 |
| Vagina | 1 | | | | 1 |
| Vagina | 1 | | | | 1 |
| Vagina | 1 | | | | 1 |
| Vagina | 1 | | | | 1 |
| Vagina | 1 | | | | 1 |
| Vagina | 1 | | | | 1 |
| Vagina | 1 | | | | 1 |
| Vagina | 1 | | | | 1 |
| Dk | | | | | |
| Dk | | | | | |
| Dk | | | | | |

The decision was made by both researchers to accept “opening” as correct, on the basis of the methodology, but this was subject to discussion.

Subgrouping discussion included grouping opening and introitus, and grouping both of these categories within the “vagina” category

Discussion regarding whether to make a “DK” subgroup, but decision made to report these separately from the blank responses but not to form a theme.

Body Part F: Perineum

| Individual responses | Emergent grouping | | | | | Judged correct by both researchers |
|---|---|---|---|---|---|---|
| | perineum | vulva | Skin at back of vagina | gooch | other | |
| Perineum | 1 | | | | | 1 |
| Perineum | 1 | | | | | 1 |
| Perinem | 1 | | | | | 1 |
| peritoneum | | | | | 1 | 0 |
| Anus | | | | | 1 | 0 |
| Vagina | | | | | 1 | 0 |
| pereneum | 1 | | | | | 1 |
| perienum | 1 | | | | | 1 |
| Perineum | 1 | | | | | 1 |
| Bartholins gland | | | | | 1 | 0 |
| perineum | 1 | | | | | 1 |
| Perinem | 1 | | | | | 1 |
| perineum | 1 | | | | | 1 |
| Perineum | 1 | | | | | 1 |
| Perineum | 1 | | | | | 1 |
| perineum | 1 | | | | | 1 |
| perrineam | 1 | | | | | 1 |
| perineum | 1 | | | | | 1 |
| vulva | | | | | 1 | 1 |
| Skin at back of vagina | | | | | 1 | 1 |
| gooch | | | | | 1 | 1 |
| Part of vagina | | | | | 1 | 0 |
| Don't know | | | | | | |
| No idea | | | | | | |
| Unknown | | | | | | |
| dk | | | | | | |
| Not sure | | | | | | |
| Dk | | | | | | |
| Dk | | | | | | |

Discussion regarding whether to make a “DK” subgroup but decision made to report these separately from the blank responses but not to form a theme.

Discussions regarding accepting vulva, skin at back of vagina and part of vagina

Body part G: Anus

| Individual responses | Emergent grouping | | | Judged correct by both researchers |
|---|---|---|---|---|
| | anus | Bottom hole | other | |
| Anus | 1 | | | 1 |
| Anus | 1 | | | 1 |
| Anus | 1 | | | 1 |
| Anus | 1 | | | 1 |
| Anus | 1 | | | 1 |
| Anus | 1 | | | 1 |
| Anus | 1 | | | 1 |
| Anus | 1 | | | 1 |
| Anus | 1 | | | 1 |
| Anus | 1 | | | 1 |
| Anus | 1 | | | 1 |
| Anus | 1 | | | 1 |
| Anus | 1 | | | 1 |
| Anus | 1 | | | 1 |
| Anus | 1 | | | 1 |
| Anus | 1 | | | 1 |
| Anus | 1 | | | 1 |
| Anus | 1 | | | 1 |
| Anus | 1 | | | 1 |
| Anus | 1 | | | 1 |
| Anus | 1 | | | 1 |
| Anus | 1 | | | 1 |
| Anus | 1 | | | 1 |
| Anus | 1 | | | 1 |
| Anus | 1 | | | 1 |
| Anus | 1 | | | 1 |

| | | | | |
|---|---|---|---|---|
| Anus | 1 | | | 1 |
| Anus | 1 | | | 1 |
| Anus | 1 | | | 1 |
| Anus | 1 | | | 1 |
| Anus | 1 | | | 1 |
| Anus | 1 | | | 1 |
| Anus | 1 | | | 1 |
| Anus | 1 | | | 1 |
| Anus | 1 | | | 1 |
| Anus | 1 | | | 1 |
| Anus | 1 | | | 1 |
| Anus | 1 | | | 1 |
| Rectum/anus | 1 | | | 1 |
| Bottom hole | | 1 | | 1 |
| Bum hole | | 1 | | 1 |
| Bottom hole | | 1 | | 1 |
| bumhole | | 1 | | 1 |
| Bum hole | | 1 | | 1 |
| mole | | | 1 | 0 |
| perineum | | | 1 | 0 |
| Dk | | | | |
| Not sure | | | | |
| Don't know | | | | |

Discussion regarding whether to make a "DK" subgroup, but decision made to report these separately from the blank responses but not to form a theme.

Decision made to record bottom/ bum hole separately to anus.

Coding Matrix Part 2B responses. N=80 participants

Note that fragments provided by an individual response could be coded to multiple groups, where relevant.

Question 2Bi: What would you call this part of the body when speaking with a romantic or sexual partner?

| Individual responses | Emergent grouping | | | | |
|---|---|---|---|---|---|
| | vagina | No words used | Vulgar or “swear word” | Female parts/ bits | “Down below” |
| Vagina | 1 | | | | |
| Vagina | 1 | | | | |
| Vagina | 1 | | | | |
| vagina | 1 | | | | |
| Vagina, sex | 1 | | | | |
| Vagina or downstairs | 1 | | | | 1 |
| Vagina, vag, private part | 1 | | | 1 | |
| I would probably use proper words as much as I knew them | | | | | |
| Not sure | | 1 | | | |
| None | | 1 | | | |
| No words | | 1 | | | |
| Pussy | | | 1 | | |
| Pussy | | | 1 | | |
| Fanny | | | 1 | | |
| cunt | | | 1 | | |
| Lips, fanny, ladygarden | | | 1 | 1 | |
| Wee warm place | | | | | |
| Girly bits | | | | 1 | |
| Lady parts | | | | 1 | |
| Girl bits, "down below", genitalia - it really depends very much on the context | | | | 1 | 1 |
| Private parts | | | | 1 | |
| bits | | | | 1 | |
| Last bits | | | | 1 | |
| Front bits, down below | | | | 1 | 1 |
| Down there | | | | | 1 |

| | | | | | |
|---|---|---|---|---|---|
| Down below or I wouldn’t use words, it would be too uncomfortable | | 1 | | | 1 |

The table shows the end result of a process of individual researcher familiarisation, coding, generating groups and subgroups, and follows a meeting between the two independent researchers to achieve consensus. The “female parts/bits” group was discussed as to whether to split into “bits”, as in fragments or pieces, and “female” but given low numbers with overlapping codes, was kept as a single group.

Question 2Bii: What would you call this part of the body when speaking with a parent or caregiver as a child?

| Individual responses | Emergent grouping | | | | |
|---|---|---|---|---|---|
| | Never discussed | Bottom/ Bum | Words only understood within the family | Private parts | Vagina/ anatomical term |
| Usually I used words from our native language which was not English, I don't really remember using any English words, we didn't talk about it much | 1 | | 1 | | |
| N/A | 1 | | | | |
| Not a subject I ever talked about with my mother, nor she with me. She gave me a book to read when I left Junior school entitled | 1 | | | | |

| | | | | | |
|---|---|---|---|---|---|
| "You're a young lady now" by Lilia White. End of sex education at age 11. | | | | | |
| Bum | | 1 | | | |
| Bum | | 1 | | | |
| Bum | | 1 | | | |
| Front bottom | | 1 | | | |
| Front bottom | | 1 | | | |
| Front bottom | | 1 | | | |
| Bottom | | 1 | | | |
| bottom | | 1 | | | |
| Front bum | | 1 | | | |
| Privates | | | | 1 | |
| Privates | | | | 1 | |
| Private parts | | | | 1 | |
| Private parts | | | | 1 | |
| Private parts | | | | 1 | |
| Private parts | | | | 1 | |
| Private parts | | | | 1 | |
| Private parts or "tute" | | | 1 | 1 | |
| Fanny, private part | | | | 1 | |
| genitals | | | | | 1 |
| groin | | | | | 1 |
| Vagina | | | | | 1 |
| Vagina | | | | | 1 |
| vagina | | | | | 1 |
| Lady parts | | | | | |
| Lady parts | | | | | |

| | | | | | |
|---|---|---|---|---|---|
| Nunny/ front bottom | | 1 | 1 | | |
| Loo loo | | | 1 | | |
| V | 1 | | | | 1 |
| Fairy | | | 1 | | |
| twinkle | | | 1 | | |
| twinkle | | | 1 | | |

Question 2Biii: What would you call this part of the body when speaking with a healthcare professional?

| Individual responses | Emergent grouping | | | | | | |
|---|---|---|---|---|---|---|---|
| | vagina | genitals | Uncertain what word to use | Female parts/ bits | Private parts | vulva | Other scientific terms |
| Vagina (34 responses) | 1 | | | | | | |
| I would probably still say vagina | 1 | | | | | | |
| Virgina | 1 | | | | | | |
| Vagina, vulva, labia | 1 | | | | | 1 | 1 |
| Vagina, genitalia (3 responses) | 1 | 1 | | | | | |
| Vulva, vagina, perineum, genitalia | 1 | 1 | | | 1 | 1 | 1 |
| If I knew the exact word and where the exact problem lay, I'd use it, otherwise say eg fanny lips or inside my vagina | 1 | | 1 | 1 | | | 1 |
| Genitals (4 responses | | 1 | | | | | |
| Genital area | | 1 | | | | | |
| Genitalia | | 1 | | | | | |
| Scientific terms | | | | | | | 1 |
| I would use the proper words if I knew them but for the ones I don't I might try to descrive what I am refering to | | | 1 | | | | 1 |
| I would use specific words | | 1 | | | | | 1 |

| | | | | | | | |
|---|---|---|---|---|---|---|---|
| for each body part or genitals as a general term | | | | | | | |
| Reproductive organs Cervix (during cervical exams) | | | | | | | 1 |
| Bum hole | | | | | | | |
| I would use formal words depending on what part I needed to discuss eg vulva, anus etc. | | | | | | 1 | 1 |
| Bum | | | | | | | |
| Vulva (4 responses) | | | | | | 1 | |
| Not sure | 1 | | | | | | |
| Don't know | 1 | | | | | | |
| Don't know- I would avoid using a word and hint at it | 1 | | | | | | |
| I have no idea what the collective name for these body parts would be, so I'd probably just point and look embarrassed. | 1 | | | | | | |
| Female parts | | | | 1 | | | |
| Female area | | | | 1 | | | |
| Privates (2 responses) | | | | | 1 | | |
| Private parts | | | | | 1 | | |

| | | | | | | | |
|---|---|---|---|---|---|---|---|
| I might say private area, I don't know. | | | | | 1 | | |

Free text comments. Further coding information.

All free text responses:

Trying to have conversations about sexual desire/dysfunction with diabetes HCPs is challenging because they really don't appear to be comfortable discussing anything beyond HbA1c, Time in Range or lipid profiles.

Unless its planning a pregnancy. And even that is mostly HbA1c and Time in Range even though that ship has sailed... Loss of libido appears to be a taboo subject along with mental health, diabetes distress and eating disorders

I have discussed sex with my diabetes consultant, mainly from a contraception point of view but I did end up discussing my sexual problems with her as a result of this and she was very supportive. I would never have considered talking about it before that appointment and I only did so because I was unsure about whether I could take contraception as a diabetic. I was also unaware that diabetes can affect my sex life.

The question about is it normal not to enjoy sex as women get older, I don't think it is normal as in right/correct. But it is very common

I have had no interest in sex since my menopause. I find sex very painful.
I would have liked her but was never offered this and am told I am now too old to start on her.

I think my husband would like to still have sex so it's a shame no one ever has spoken to me about this.

I haven’t thought about this being connected with diabetes before

Our knowledge of our own sexual system and health is very fragmented. Unless you educate yourself, noone discusses it, it is a taboo. Yet it should be talked about so we can take better care of ourselves, know the difference between parts and understand better our functioning. It all starts with education!

I have never had any discussion about my sexual health in over 50yrs living with T1D. I have only seen female consultants in the last 9yrs

I have had sexual issues in the past surrounding extreme pain when trying to have sex for the first time. I was aware that this may have been linked to my diabetes but I still did not choose to address it with my diabetes team, instead I went to a private gynaecologist. I think this shows that I did not feel that my diabetes team would have been able to help me. The only time I have spoken to my diabetes team about sexual issues was to ask if there were any issues with getting the implant for hormonal contraception and whether it would affect my blood sugar levels.

Never been asked about sexual matters at diabetic reviews. My DN would fall off her chair if I brought it up.

I’ve had to look up online for answers in regards to sexual health problems combined with diabetes.  My diabetes nurse didn’t seem to have any information on this, which was disappointing/worrying.

I have no access to specialist diabetes support so I’d be ecstatic to talk about anything at all if it meant I got an appointment.

I’ve spoken about sex connected to contraception and planning pregnancy but not about just sex for its own sake

I had never thought about this before. I have been asked about my sex life to do with avoiding getting pregnant without planning it for my diabetes but no one has spoken about my sex life and diabetes otherwise.
It is hard enough starting relationships without managing diabetes as well.

I have never had a diabetes health professional ask my about my sexual health or sex life. Currently I don't have a lot of confidence in my diabetes team that they could handle the topic very well, but I'm sure there are diabetes healthcare professionals that would be understanding and I would be comfortable speaking to about this if I was under someone else's care.
Usually my diabetes team barely gets through asking me about hypos and trying (and failing) to download my glucose readings, and it doesn't feel like the most competent team. If I had a problem, I don't feel like they would be the ones to help me.
My GP practice is quite good, I don't have a consistent GP but everyone there has been professional and I feel like I could speak with them if I had an issue. I think this is important research, I look forward to hearing more about it.
Create awareness about diabetes and his harmfulness
I'd be mega surprised if anyone at a diabetes clinic ever raised the subject of anything sexual, though I know that some ladies do have BG problems around their monthly cycles and obviously during pregnancy

Fragments grouped by theme:

In order to arrive at this grouping, a reflective thematic process was followed by two independent researchers, and involved coding fragments using MAXQDA software. Groupings and themes/ subthemes emerged. The two researchers met to discuss the groupings and reach consensus on themes emerging from the data. The main themes are below and coded fragments within the theme groupings are provided. Further data on the coding process is available on request.

Theme 1: Diabetes HCPs' priority is blood sugar management.

Coded fragments:

"Trying to have conversations about sexual desire/dysfunction with diabetes HCPs is challenging because they really don't appear to be comfortable discussing anything beyond HbA1c, Time in Range or lipid profiles."

"The only time I have spoken to my diabetes team about sexual issues was to ask if there were any issues with getting the implant for hormonal contraception and whether it would affect my blood sugar levels."

"I'd be mega surprised if anyone at a diabetes clinic ever raised the subject of anything sexual, though I know that some ladies do have BG problems around their monthly cycles and obviously during pregnancy"

"Usually my diabetes team barely gets through asking me about hypos and trying (and failing) to download my glucose readings,"

Theme 2: Diabetes HCPs discuss sex in connection to contraception and planning pregnancy rather than to promote positive sexual health or sexual enjoyment.

Coded fragments:

"Trying to have conversations about sexual desire/dysfunction with diabetes HCPs is challenging because they really don't appear to be comfortable discussing anything beyond HbA1c, Time in Range or lipid profiles. Unless its planning a pregnancy"

"I have discussed sex with my diabetes consultant, mainly from a contraception point of view"

“The only time I have spoken to my diabetes team about sexual issues was to ask if there were any issues with getting the implant for hormonal contraception and whether it would affect my blood sugar levels”

“I’ve spoken about sex connected to contraception and planning pregnancy but not about just sex for its own sake”

“I have been asked about my sex life to do with avoiding getting pregnant without planning it for my diabetes but no one has spoken about my sex life and diabetes otherwise. It is hard enough starting relationships without managing diabetes as well.”

Theme 3: Lack of awareness of a link between diabetes and sexual problems

Coded fragments:

“I was also unaware that diabetes can affect my sex life.”

“I haven’t thought about this being connected with diabetes before”

“Our knowledge of our own sexual system and health is very fragmented.”

“I’ve had to look up online for answers in regards to sexual health problems combined with diabetes.  My diabetes nurse didn’t seem to have any information on this, which was disappointing/worrying.”

“I had never thought about this before”

Theme 4: Silence/ taboo regarding communication about sex during diabetes care.

Coded fragments:

“Unless you educate yourself, noone discusses it, it is a taboo”

“I have never had any discussion about my sexual health in over 50yrs living with T1D.”

“Never been asked about sexual matters at diabetic reviews. My DN would fall off her chair if I brought it up.”

“Loss of libido appears to be a taboo subject along with mental health, diabetes distress and eating disorders”

“I'd be mega surprised if anyone at a diabetes clinic ever raised the subject of anything sexual”

3. Supplementary Table S1: Main Survey Question Responses (Results presented in Table 2, expressed as number and percentage of respondents per question).

| Question Stem | Response Options (% of Participants who selected an answer to the question) | | | | | |
|---|---|---|---|---|---|---|
| | Strongly Agree | Somewhat Agree | Neither Agree nor Disagree | Somewhat Disagree | Strongly Disagree | Don't Know |
| 1. At a diabetes check-up, if a health professional asks about my sex life, I would be surprised.<br>Total question responses: 143<br>Blank responses: 20 | 88 (61.5%) | 26 (18.2%) | 11 (7.7%) | 9 (6.3%) | 7 (4.9%) | 2 (1.4%) |
| 2. I have discussed my sex life at a diabetes check up in the past.<br>Total question responses: 139<br>Blank responses: 24 | 17 (12.2%) | 27 (19.4%) | 9 (6.5%) | 20 (14.4%) | 65 (46.8%) | 1 (0.7%) |
| 3. I feel well-informed about how diabetes can affect women's sex lives.<br>Total question responses: 157<br>Blank responses: 6 | 21 (13.4%) | 21 (13.4%) | 10 (6.4%) | 25 (15.9%) | 66 (42.0%) | 14 (8.9%) |
| 4. A healthcare professional has told me women with diabetes are at increased risk of having problems with their sex lives.<br>Total question responses: 141<br>Blank responses: 22 | 22 (15.6%) | 11 (7.8%) | 11 (7.8%) | 19 (13.5%) | 73 (51.8%) | 5 (3.5%) |
| 5. I have been advised who to speak to about my sex life if I have any concerns.<br>Total question responses: 146<br>Blank responses: 17 | 12 (8.2%) | 16 (11.0%) | 13 (8.9%) | 19 (13.0%) | 83 (56.8%) | 3 (2.1%) |

| 6. | Response options | Number of responses (% of all participants) |
|---|---|---|

| | | |
|---|---|---|
| If I had a problem with my sex life, the healthcare professional I'd talk to first is....<br><br>Total responses 128<br>Blank responses: 35 | GP | 44 (34.4%) |
| | Practice nurse | 9 (7.0%) |
| | Walk in sexual health clinic | 20 (15.6%) |
| | Diabetes secondary care team professional | 18 (14.1%) |
| | I probably would not speak to a health professional at all | 22 (17.2%) |
| | This situation would never be relevant to me | 2 (1.6%) |
| | Don't know | 11 (8.6%) |
| | Other | 2 (1.6%)<br>Free text responses: "peer support group"; "If I knew it was diabetes related, I would speak to the diabetes team, otherwise I'd speak to the GP or sexual health clinic" |

| 7.<br>If needed help with problems with my sex life, and had to make an appointment with someone I hadn't met before, I would prefer to speak to…<br><br>Total responses: 113<br>Blank responses: 50 | Response options | | | | | | |
|---|---|---|---|---|---|---|---|
| | A female HCP | A Male HCP | An HCP of any gender | Don't know | I wouldn't speak to an HCP | This subject would never be relevant to me | An HCP who identifies as non-binary or gender queer |
| | 60 (53.1%) | 5 (4.4%) | 35 (31.0%) | 4 (3.5%) | 6 (5.3%) | 1 (0.9%) | 2 (1.8%) |

| 8.<br>You have known 2 healthcare professionals well for a long time. They have the same job and are the same age.<br>You trust them both equally. They both listen well and have good knowledge.<br><br>As far as you can tell from appearance and name, one professional is a man, and one is a woman.<br><br>Please consider the following statement<br><br>"If I had problems with my sex life, I would prefer to speak to..."<br><br>Total question responses: 126 | Response options | | | | |
|---|---|---|---|---|---|
| | The female HCP | The Male HCP | Either HCP | I don't know | I wouldn't speak to an HCP |
| | 75 (59.5%) | 4 (3.2%) | 36 (28.6%) | 3 (2.4%) | 8 (6.3%) |

| | Response options | | | | | |
|---|---|---|---|---|---|---|
| Blank responses: 37 | | | | | | |
| 9.<br>For me personally:<br><br>At a diabetes check-up, if I am offered information about sex and diabetes and the option to discuss this topic, I will feel pleased.<br><br>Total question responses: 130<br>Blank responses: 33 | Strongly Agree | Somewhat agree | Neither agree nor disagree | Somewhat disagree | Strongly disagree | Don't know |
| | 47 (36.1%) | 41 (31.5%) | 22 (16.9%) | 10 (7.7%) | 4 (3.1%) | 6 (4.6%) |
| 10.<br>For me personally:<br><br>At a diabetes check-up, if I am offered information about sex and diabetes and the option to discuss this topic, I will feel upset.<br><br>Total question responses: 132<br>Blank responses: 31 | 12 (9.1%) | 6 (4.5%) | 9 (6.8%) | 18 (13.6%) | 84 (63.6%) | 3 (2.3%) |
| 11.<br>For me personally:<br><br>At a diabetes check-up, if I am offered information about sex and diabetes and the option to discuss this topic, I will feel offended.<br><br>Total question responses: 135<br>Blank responses: 28 | 5 (3.7%) | 9 (6.7%) | 7 (5.2%) | 6 (4.4%) | 105 (77.8%) | 3 (2.2%) |
| 12.<br>For me personally:<br><br>At a diabetes check-up, if I am offered information about sex and diabetes and the option to discuss this topic, I will feel embarrassed.<br><br>Total question responses: 138<br>Blank responses: 25 | 11 (8.0%) | 30 (21.7%) | 16 (11.6%) | 27 (19.6%) | 49 (35.5%) | 5 (3.6%) |
| 13.<br>For me personally:<br><br>At a diabetes check-up, if I am offered information about sex and diabetes and the option to discuss this topic, I might mention | 35 (26.5%) | 54 (40.9%) | 12 (9.1%) | 11 (8.3%) | 14 (10.6%) | 6 (4.5%) |

| | | | | | | |
|---|---|---|---|---|---|---|
| problems I haven't spoken to healthcare professionals about yet.<br><br>Total question responses: 132<br>Blank responses: 31 | | | | | | |
| 14.<br>For me personally:<br><br>At a diabetes check-up, if I am offered information about sex and diabetes and the option to discuss this topic, I would feel forced to discuss something I'd rather not talk about.<br><br>Total question responses: 151<br>Blank responses: 12 | 6<br>(4.0%) | 17<br>(11.3%) | 16<br>(10.6%) | 16<br>(10.6%) | 92<br>(60.9%) | 4<br>(2.6%) |
| 15.<br>I expect the health professionals I see at diabetes check-ups are trained about diabetes and women's sexual problems.<br><br>Total question responses: 156<br>Blank responses: 7 | 72<br>(46.2%) | 42<br>(26.9%) | 13<br>(8.3%) | 9<br>(5.8%) | 9<br>(5.8%) | 11<br>(7.1%) |
| 16.<br>If I start talking about my sex life at a diabetes check-up, I expect the health professional will feel pleased.<br><br>Total question responses: 129<br>Blank responses: 34 | 11<br>(8.5%) | 18<br>(14.0%) | 23<br>(17.8%) | 23<br>(17.8%) | 46<br>(35.7%) | 8<br>(6.2%) |
| 17.<br>If I start talking about my sex life at a diabetes check-up, I expect the health professional will think the topic is inappropriate.<br><br>Total question responses: 136<br>Blank responses: 27 | 38<br>(27.9%) | 21<br>(15.4%) | 21<br>(15.4%) | 15<br>(11.0%) | 29<br>(21.3%) | 12<br>(8.8%) |
| 18.<br>At a diabetes check-up, my sex life is a high priority for the health professional.<br><br>Total question responses: 123<br>Blank responses: 40 | 10<br>(8.1%) | 13<br>(10.6%) | 11<br>(8.9%) | 14<br>(11.4%) | 70<br>(56.9%) | 5<br>(4.1%) |

| | | | | | | |
|---|---|---|---|---|---|---|
| 19.<br>At a diabetes check-up the healthcare professional has time to discuss my sex life.<br><br>Total question responses 133<br>Blank responses: 30 | 12<br>(9.0%) | 15<br>(11.3%) | 18<br>(13.5%) | 27<br>(20.3%) | 54<br>(40.6%) | 7<br>(5.3%) |
| 20.<br>If I start talking about my sex life at a diabetes check-up, I expect the healthcare professional will feel annoyed.<br><br>Total question responses: 109<br>Blank responses: 54 | 25<br>(22.9%) | 19<br>(17.4%) | 11<br>(10.0%) | 19<br>(17.4%) | 30<br>(27.5%) | 5<br>(4.6%) |
| 21.<br>If I start talking about my sex life at a diabetes check-up, I expect the healthcare professional will feel embarrassed.<br><br>Total question responses: 128<br>Blank responses: 35 | 14<br>(10.9%) | 15<br>(11.7%) | 23<br>(18.0%) | 24<br>(18.8%) | 42<br>(32.8%) | 10<br>(7.8%) |
| 22.<br>I think good treatments are available on the NHS if women experience problems with their sex lives.<br><br>Total question responses: 146<br>Blank responses: 17 | 11<br>(7.5%) | 15<br>(10.3%) | 13<br>(8.9%) | 24<br>(16.4%) | 44<br>(30.1%) | 39<br>(26.7%) |
| 23.<br>It's normal for women to have less enjoyable sex lives as they get older.<br><br>Total question responses: 135<br>Blank responses: 28 | 29<br>(21.5%) | 39<br>(28.9%) | 17<br>(12.6%) | 16<br>(11.9%) | 17<br>(12.6%) | 17<br>(12.6%) |
